#  A Neuro-Computational Model for Discrete-Continuous Dual-Task Process

**DOI:** 10.3389/fncom.2022.829807

**Published:** 2022-03-29

**Authors:** Maryam Sadeghi Talarposhti, Mohammad Ali Ahmadi-Pajouh, Farzad Towhidkhah

**Affiliations:** Department of Biomedical Engineering, Amirkabir University of Technology (Tehran Polytechnic), Tehran, Iran

**Keywords:** dual-task, modeling, attention, Van der Pol oscillator, synchronization, Parkinson's, ADHD

## Abstract

Studies on dual-task (DT) procedures in human behavior are important, as they can offer great insight into the cognitive control system. Accordingly, a discrete-continuous auditory-tracking DT experiment was conducted in this study with different difficulty conditions, including a continuous mouse-tracking task concurrent with a discrete auditory task (AT). Behavioral results of 25 participants were investigated *via* different factors, such as response time (RT), errors, and hesitations (pauses in tracking tasks). In DT, synchronization of different target neuron units was observed in corresponding brain regions; consequently, a computational model of the stimulus process was proposed to investigate the DT interference procedure during the stimulus process. This generally relates to the bottom-up attention system that a neural resource allocates for various ongoing stimuli. We proposed a black-box model based on interactions and mesoscopic behaviors of neural units. Model structure was implemented based on neurological studies and oscillator units to represent neural activities. Each unit represents one stimulus feature of task concept. Comparing the model's output behavior with the experiment results (RT) validates the model. Evaluation of the proposed model and data on RT implies that the stimulus of the AT affects the DT procedure in the model output (84% correlation). However, the continuous task is not significantly changed (26% correlation). The continuous task simulation results were inconsistent with the experiment, suggesting that continuous interference occurs in higher cognitive processing regions and is controlled by the top-down attentional system. However, this is consistent with the psychological research finding of DT interference occurring in response preparation rather than the stimulus process stage. Furthermore, we developed the proposed model by adding qualitative interpretation and saving the model's generality to address various types of discrete continuous DT procedures. The model predicts a justification method for brain rhythm interactions by synchronization, and manipulating parameters would produce different behaviors. The decrement of coupling parameter and strength factor would predict a similar pattern as in Parkinson's disease and ADHD disorder, respectively. Also, by increasing the similarity factor among the features, the model's result shows automatic task performance in each task.

## Introduction

Humans can regularly perform several tasks simultaneously in their daily lives. However, it appears that the human mind faces difficulties when performing two tasks simultaneously, occasionally failing to exhibit proper output behaviors (Telford, 1931). Studies have shown that performing dual-task (DT) involves more limitations compared to performing a single task (ST; Welford, 1952; McCann and Johnston, 1992; Yogev et al., 2005; Sigman and Dehaene, 2006; Kiesel et al., 2010; Klapp et al., 2019). An ST may consist of several stages before achieving a response, such as stimulus perception, response selection, and execution; meanwhile, in the DT condition, the time coordination of several task features would be added to this complexity (Pashler, 1994; Meyer and Kieras, 1997; Pashler et al., 2000). The challenging aspect of the DT condition often results in impairments in one or both tasks, causing longer response times (RTs) and more errors (Telford, 1931; Welford, 1952; Kahneman, 1973; McCann and Johnston, 1992; Schubert et al., 2008; Ewolds et al., 2017).

Considering a task as a paradigm consisting of three parts, namely, input, perceptual process, and output (Kiesel et al., 2010), various stages of DT performance analysis with different features can reveal the limitations and potentials of human cognitive and motor systems (Buss et al., 2014; Ewolds et al., 2017). There have been behavioral responses to the challenging aspect of the DT condition in many neurodegenerative diseases. For example, stride signal has severely deteriorated in patients with Parkinson's disease while walking along with the n-back experiment (Yogev et al., 2005; Bazanova et al., 2018; Möhring et al., 2018). The variability of stride signals and fall risk were shown to be higher under DT conditions (Hung et al., 2020; Kahya et al., 2020; Penko et al., 2020; Mishra and Thrasher, 2021). Also, research studies on children with attention deficit hyperactivity disorder (ADHD) indicated more problems in children with ADHD while performing gait tasks concurrent with hearing and memorizing numbers (Manicolo et al., 2017). Similar results were observed for children with ADHD under oculomotor and postural DT conditions (Caldani et al., 2019).

Despite numerous differences between the DT and ST conditions, the sensory processing stage (i.e., the first stage of the task process) in DT and ST is considerably similar. This stage receives information from the associated device or simply the environment (Manor et al., 2016). Then, the bottom-up attentional system attempts to connect the sensory clue to the bold pre-defined response features (e.g., color, movement, appearance, etc.) (McCann and Johnston, 1992). The sensory information can be obtained from different sensory sources, such as visual, verbal, and movement as well as response presenting output (Kiesel et al., 2010). When a DT condition operates, one or more sensory inputs may appear simultaneously, possibly causing cross-talk interference (Kinsbourne, 1981; Swinnen and Wenderoth, 2004). This typically occurs when both sensory inputs use same neuronal streams (e.g., two vocal or two visual inputs that overlap; Pashler, 1994; Meyer and Kieras, 1997; Schubert, 1999; Schubert et al., 2008; Duncan et al., 2021). The DT interference examined in this study is defined as two different streams of sensory inputs that take place at the same time, requiring simultaneous motor responses. Such a definition necessitates the DT process to follow the bottleneck theory (Welford, 1967; McCann and Johnston, 1992).

In brief, the bottleneck theory implies that there is a central attentional limitation that does not allow the processes of two tasks to proceed (Pashler, 1994). The reason behind this limitation has been associated with the limitation of the central processor and memory. Therefore, in the DT condition, two tasks cannot be processed at the same time, which, in turn, creates bottleneck interference (Welford, 1952; Netick and Klapp, 1994; Schubert, 2008; Reimer et al., 2015).

There have been several hypotheses as to what causes DT interference. In this regard, Netick and Klapp (1994) designed an experiment to analyze DT in different stages (Netick and Klapp, 1994); they defined three process stages, namely, stimulus perception, response preparation, and response execution. Stimulus perception refers to the comprehension of a stimulus and relating it to a response. Response preparation consists of the processes before response execution. The response execution stage refers to a movement that occurs to generate a response (Netick and Klapp, 1994; Watter and Logan, 2006). Their experiment consisted of an auditory stimulus-based response and a pursuit-tracking (PT) task using a joystick displayed on a screen (continuous task). The continuous task would continuously be executed when discrete auditory stimuli appear; in other words, participants must not stop the PT as they simultaneously respond to the auditory stimulus. The details of this experiment are elaborated in the method section. To reach this aim, they used the analysis results of hesitations that happened during the PT task in the DT condition with the auditory task (AT). Observations showed that responding to the AT while performing PT leads to pauses in PT performance, which freezes the position for a short period. These pauses were measured and called “hesitations” (H). H occurrence is somehow associated with RT appearance in the discrete task; therefore, both RT and H are time-based representatives of DT interference. Accordingly, different ATs were implemented to examine the limitations of this new factor and investigate how H varies at a different level of AT difficulties (Netick and Klapp, 1994). However, there were some gaps in their experiment. Importantly, no changes were made in the PT task throughout the entire experiment. As a follow-up to this research, the experiment was repeated in this study with added difficulty in the PT task, based on manipulations made on the target area. This was done to observe if the interference would be affected by PT task difficulty level or if it is a variable that depends on AT difficulty.

According to the results of the analysis by Netick and Klapp (1994), the interference of discrete-continuous DT condition occurs in the response preparation stage as opposed to the stimulus perception and execution stages based on analysis of the H factor in PT task (Netick and Klapp, 1994). This study would repeat their experiment with different difficulty levels added to the PT task while using a behavioral black box synchronization-based model to interpret the stimulus perception stage.

### Dual-Task Models in Prior Research Studies

The attention system is divided into bottom-up and top-down controllers. The role of the bottom-up controller is to analyze sensory input and stimuli that happen involuntarily. This role requires the bottom-up attention to be sensitive to the stimuli's features. The bottom-up process is fast and involves a low level of the sensory region activated in the brain, such as the occipital and temporal lobes for visual and auditory stimuli, respectively. Contrary to the bottom-up controller, the top-down controller's role is to recognize predefined targets and aims as an attentional system that activates voluntarily; therefore, it involves delays similar to all voluntary actions. The top-down attention activates higher levels of cognitive-based brain regions, such as the prefrontal lobe (Katsuki and Constantinidis, 2013; Ramirez-Moreno et al., 2013).

Studies show that irrelevant signals and distractors in the environment may cause false alarms for sensory information processing, resulting in misleading interpretations, wrong responses, or delays in behaviors (Gazes et al., 2010; Parmentier et al., 2011). In this situation, what would happen if there was a second target that should be processed alongside the first one? As mentioned earlier, the processing of an additional task would be postponed by the bottleneck until the first one is processed. On the other hand, there can be a competition among bottom-up units to respond to the second stimulus. Notably, since this competition becomes an attentional demand process, higher processing resources can be affected by this challenge among tasks. Certain studies have demonstrated that when DT is performed alongside relevant regions of two tasks in the brain, the dorsolateral prefrontal cortex is activated remarkably (Buss et al., 2014; Heinzel et al., 2017; Kimura et al., 2021; Mishra and Thrasher, 2021). This phenomenon has been observed in different types of DT conditions. Therefore, it appears that this region may have been activated because of competition between two tasks (Heinzel et al., 2017; Ljubisavljevic et al., 2019). Consequently, understanding how bottom-up attention handles competition features is of substantial importance. Since this competition for highlighting targets is associated with higher processing regions, such as the parietal and prefrontal lobes, this can be considered as activities, including synchronization and desynchronization (Gross et al., 2004; Bridwell and Srinivasan, 2012). Besides, previous studies have shown that the superior parietal and parieto-occipital regions are activated in pursuit of tracking tasks (Hill, 2014; Kobler et al., 2018).

The “adaptive resonance theory,” which is applied in investigations on learning and memory, can be taken into account to explain synchronization as well (Proulx and Egeth, 2006). Based on this theory, the neural procedure consists of the attentional demand dilemma, synchronization, and resonance. Here, dilemma means requiring a type of stability that is flexible for new information and preserving the old ones (Mermillod et al., 2013). Consequently, there would be a trade-off between stability and plasticity in this case. As a result, synchronization may be a key factor, given its modulatory effect on sensory input processing. The concept of top-down and bottom-up attention has been investigated in Baghdadi's model with respect to the oscillatory behavior for features of sensory input. This model represented the relationship between top-down and bottom-up attention through various Van der Pol oscillators based on synchronization and desynchronization (Baghdadi et al., 2019).

In this study, a DT black-box model is proposed based on Baghdadi's model (Baghdadi et al., 2019) structure. The proposed model explains several aspects of the behavioral observation related to bottom-up attention as well as a number of interpretations regarding top-down attention. It is important that the model does not take the structure of brains and functionality into account. Also, the behavioral-acquired data are obtained and compared to model results to validate the mesoscopic proposed model. However, it was attempted to consider the oscillatory nature of the neuronal system and characteristics of the experiment. The results of the proposed model also reveal information about the nature of hesitation, which is a type of error in the continuous task.

In the prediction section, the proposed model's generality is investigated by parameter manipulations. The comparison of neural unit's activities with ERD and ERP signals is generally described in qualitative method and focused on differences in alternations of patterns. Based on previous studies, the ERP signal unfolds some behavioral components in peaks and latencies like P300 (Gerson et al., 2005; Kida et al., 2012). Also, the amplitude of ERP signals can reveal some features of behavioral results. For example, the longer the time between two stimuli, the more significant the peak of P300 would happen in the ERP signal, so the stimuli's properties would affect the amplitude of ERP peaks (Mugruza-Vassallo and Potter, 2019). In addition, the difficulty of visual and auditory stimuli and the required response's complexity have been shown to be effective in gamma-band activity (Gundel and Wilson, 1992; Lachaux et al., 2005; Mulert et al., 2010).

The information on ERP can be interpreted as behavioral information, such as latencies associated with response time and alternations (amplitudes and frequencies), which reveal deeper information on brain activities (Keil et al., 2001). Therefore, extracting behavioral features from ERP signals is not inconsistent with the general idea of the model.

On the other hand, a critical aspect of this model is that we used the concept of threshold. The threshold can be action potential, the level of dopamine (Guthrie et al., 2009), or any measurable physiological activity that triggers a function. Therefore, using the black-box model, we defined the output signal as “activity,” which is not precisely the action potential and is scalable for any known physiological component that can have effective levels in functionality. Therefore, one contribution of such a model can be the ERP signal's components, as explained earlier. Predictions generally showed effectiveness in investigating motor problems in certain neurodegenerative diseases, such as Parkinson's disease, and attentional deficit disorders, such as ADHD.

## Methods

Here, we will explain the details of the experiment and then the structure of the proposed model.

### Experiments

In this section, Details of the experiment are elaborated. As mentioned earlier, the experiment was based on the study by Netick and Klapp (1994), and we added two different difficulties in the PT task by changing the target's area, and the experiment was conducted with a mouse rather than a joystick.

#### Participants

A total of 25 healthy subjects with normal hearing and motor responses participated in the experiment [10 male and 15 female subjects with age range of 20–35; Mean (M) = 27, SD = 4.5]. The experiment was performed under the approval of the Psychology Department Laboratory of the Martin Luther Halle University of Germany and under the supervision of Prof. Torsten Schubert.^1^ The subjects were all healthy and right-handed people; in addition, they were all Martin Luther students who took part in the study as volunteers and received either course credits or 8 euros/h as payment for completing the experiment. Importantly, they were informed about the concept of the experiment and were asked to maintain the positions of their hands and chairs still during the entire experiment period, except for break times. The experiment consisted of two types of tasks, the auditory task (AT) and the pursuit-tracking (PT) task, displayed on the screen.

#### The Auditory Task Experiment

##### Setup

The AT experiment was entirely performed with the same setup: The participants listened to different tones constructed by the programming software MATLAB (ver. 2009b) in four different sound frequencies played using headphones with a frequency response of 18–22,000 Hz, Dynamic Transducer principle, and sound pressure level: 110 dB (1 kHz/1 V RMS). The experiment was conducted inside a soundproof room, and the subjects were asked to select the related answers and implement motor responses on a computer system (CPU: Intel Core i5-4670, 3.4 GHz). All the participants listened to the same trials with 333 ms in randomized order. The fixed features of sound waves involved an amplitude of 5 (on a scale of 1–10) and a sampling rate (fs) of 44,100 Hz. The stimuli were characterized by pitch, i.e., recognized by sound frequency. This experiment used two different pitches, 500 and 2,000 Hz, which were regarded as low-pitched and high-pitched sounds, respectively. Two more frequencies (200 and 1,600 Hz) were used for the training section to eliminate confounding variables that interfere with stimuli recognition. The participants were asked to respond to the AT stimulus by pressing specific buttons on a keyboard (model: Logitech K120, depth/height: 23.5 +/- 1 mm). The participants were also instructed to place their left-hand fingers on four keys (A, S, D, and F) and maintain this position during the whole experiment.

##### Design

The two ATs in the task included the simple response time (SRT) task and the choice response time (CRT) task. Both ATs were established and conducted similar to the study by Netick and Klapp (1994). Moreover, there were single ATs and dual-task conditions, in which AT and PT tasks were simultaneously performed.

###### Simple Response Time.

The simple response time (SRT) auditory task is the basic AT defined as the auditory stimulus and response time with certain features, such as sound frequency of 2,000 Hz. In this research, the participants were asked to respond to the stimulus as fast as they could by pressing the F key.

###### Choice Response Time.

The choice response time (CRT) task is described as two different responses to two different stimuli. The participants were asked to distinguish between the two stimuli. Based on given instructions, they had to press the F and D keys as fast as possible when they hear the 2,000 and 500 Hz stimuli, respectively.

#### Pursuit-Tracking Task Experiment

##### Setup

The force function^2^ of PT was established using the same computer system and software. The PT target was displayed on a 24-inch LED screen (1,920 × 1,080 @120 FPS^3^), and a mouse (optical USB mouse, 800 dpi resolution) was used for pursuit. The participants were asked to maintain their hands in a relaxed position and place their right hand on the mouse as they concentrate on the PT.

##### Design

**Figure 1:**
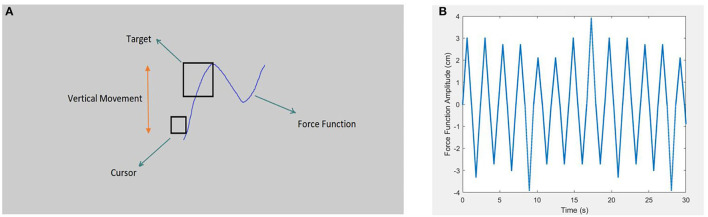
Schematic representation of the pursuit-tracking (PT) task's environment and force function curve is depicted in the right and left picture, respectively. **(A)** Shows the general screen of the PT task (not to scale). The screen background color was decided to be neutral gray to be comforting to the eyes. The schematic also presents the target (the square, 3 or 4 times larger than the cursor), and the cursor (the smaller square) is also demonstrated. Target movement was limited to the vertical axis *via* the pseudo-sinusoidal force function. The participants were asked to maintain the cursor inside the target (Sadeghi Talarposhti et al., 2020). **(B)** Shows the pseudo-sinusoidal force function of the PT task with random change in amplitude and dot's density, which determines the speed of movement. Sharp amplitudes avoid artificial hesitations in peaks that are caused by the sinusoidal force function.

As shown in Figure 1, target pursuit in PT was performed exclusively in the vertical axis, with a 2.6-mm square cursor (Netick and Klapp, 1994). As intended, the square-shaped target was 3–4 times larger than the cursor (three times larger in hard tracking and four times larger in easy tracking). The participants were instructed to keep the cursor inside the target during the task. Notably, the PT task was established with the tracking force function of the sum of different pseudo-sinusoidal frequencies. This study used different frequencies and amplitudes to offer an attention-demanding tracking task. Additionally, to avoid artificial hesitations at the sinusoidal peak, tracking speed was changed from positive to negative acceleration with regard to the peak of the sinus to the sum of different constant speeds that vary through small time intervals. As a result, the pattern was still sinusoidal in which accelerations showed a pseudo-triangle function. This change was implemented to prevent confounding force functions of natural hesitation when calculating human hesitations in DTs. Alternatively, considerable attempts were made to present the tracking as a sinusoidal function by examining different velocities in the pilot study. Accordingly, constant speed values were decided as 3.62, 4.57, 5.17, 5.5, and 6.72 cm/s, along with amplitudes of 2.1, 2.7, 3, 3.3, and 3.9 cm. We employed a random combination of force function speed and amplitudes, which offered 25 conditions that were nearly impossible for the participants to predict the following change in direction; therefore, tracking was an attention-demanding task throughout the whole experiment.

#### Experiment Procedure

We assigned three different values to the AT and PT tasks, where each value represents a different task under its sensory system's category. Accompanied by PT, AT generated eight different task combinations. The experiment procedure consisted of eight 180-s sequences (each entailing 14 AT trials) with a time interval of 10–15 s. The task combination condition remained fixed during the sequence with both essential (10 s) and optional break times between sequences. As shown in Table 1, each sequence was referred to as seq (AT, PT).

**Table 1:** Combination of auditory task (AT) and pursuit-tracking (PT).

**Tasks**	**Values**	**0**	**1**	**2**
AT		PT Single Task *(No AT included)*	SRT *(Single Reaction Time task)*	CRT *(Choice Reaction Time task)*
PT		AT Single Task *(No PT included)*	EPT *(Easy Pursuit-Tracking task)*	HPT *(Hard Pursuit-Tracking task)*

*Each AT type and PT difficulty level establish a dual/single-task condition; the conditions remained the same during the sequence, represented as seq (AT, PT). The first and second elements represent the AT and PT conditions, respectively*.

**Figure 2:**
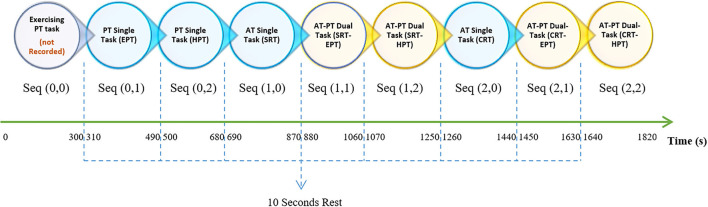
In the experiment procedure diagram, each circle shows a sequence of trails that continue for 180 s, shown as two-component matrices. Circles with blue and yellow colors represent ST and DT, respectively. In seq (AT, PT), the first and second components represent the number of AT and PT tasks, respectively. Notably, there were 10-s breaks between sequences that are shown with blue dashed lines. The sequences started with a training part, i.e., seq (0, 0), as two 150-s pieces of training that are not recorded. In addition to the PT in the training sequence, different pitch stimuli were initiated. The duration of the entire experiment was ~33 min. ST, single task; DT, dual task; AT, auditory task; PT, pursuit-tracking; seq, sequence; SRT, simple reaction time; CRT, choice reaction time; EPT, easy pursuit-tracking; HPT, hard pursuit-tracking.

As pointed out earlier, the term “sequence” refers to 180 s of performing tasks that entail STs and DTs. Sequences with a value of “0” refer to an ST. The experiment started with two training parts, both the AT and PT training, and results were not recorded for further analysis. Considering the values listed in Table 1 and the seq (AT, PT) format, sequences following training would be seq (0, 1) and seq (0, 2), which indicate the ST for EPT and HPT, respectively, as the PT control condition. Seq (1, 0) and seq (2, 0) represent ST for SRT and CRT, respectively, as the AT control condition. Other sequences with no zero in their entries indicated a DT condition. In DT conditions, the participants were asked to simultaneously perform the determined AT and PT. The order of the entire experiment is explained in Figure 2.

Consequently, the experiment consisted of six sequences, including the ST and DT tasks. Certain data were excluded, such as key press location and timing of AT task, cursor position, time of key press content, and timing in the PT task. Therefore, RT and error rates for AT tasks are generally calculable. Regarding the PT task, position information was used to calculate the time during which the cursor was outside of the target, and we reported the result as percentage of the associated sequence. This value pertains to PT error. Another analysis was conducted to calculate hesitation based on both position and timing. Pauses made by a participant during PT tasks were calculated; these were defined as duration when the cursor was fixed at one y position for longer than 333 ms.

### The Proposed Model

It was stated in the previous section that certain parts of the brain are involved in AT and PT, as well as in DT conditions. Here, one neural system can be considered to respond to stimuli (auditory and visual) following their control policies. The proposed model is based on synchronization and desynchronization behaviors inspected during responses to sensory inputs and their interactions to generate a response. Since the model was considered as a black box, precise information on activated regions of the brain was not taken into account. To this end, oscillatory units were employed to show the synchronization/desynchronization behavior as it occurs when the time scale adjusts along with oscillatory unit interactions. This adjustment is possible with forced and mutual synchronizations (Balanov et al., 2009).

Frequency or phase-locking synchronization happens between unit oscillations. As a result, one or both system unit dynamics may be suppressed. In other words, if two system frequencies are the same or there are no phase differences between them, they are synchronized; however, if the two systems oscillated with different frequencies or in different phases, desynchronization would occur. Subsequently, synchronizing two systems by external imposition of another oscillating system is possible. Force-frequency/phase synchronization can be achieved by fixating the coupling weight between two units. In this study, a Van der Pol oscillator was used to show unit synchronization or desynchronization. Notably, the Van der Pol oscillator was selected for various reasons; first, it is a popular oscillator in biological and neural modeling carried out in previous studies (Nomura et al., 1993; Hu and Chung, 2013; Euzébio and Llibre, 2016). Second, as the Van der Pol oscillator ignores details of neuronal reactions, avoiding complexities in the model would be quite suitable. Third, the oscillation of Van der Pol is easily tractable analytically by adjusting its amplitude and frequency, and simulation results can focus on the main goal of the model. However, the simplistic aspect of Van der Pol, which is an advantage in this study, can be replaced by a more complex oscillator to enhance the generality and capability of the proposed model.

The Van der pol oscillation equation is presented as a second-order differential equation [equation (1)]. Equation (1) represents the second-order Van der Pol oscillator in its general form:


(1) $$\begin{array}{l} \ddot{Y}-\;\left(\lambda -Y^{2}\right)\dot{Y}+\;p^{2}Y\;=\;0 \end{array}$$

where λ is the bifurcation parameter, and its value represents the oscillation stage; if it was lower than zero (λ ≤ 0), no oscillation would occur, and if it was higher than zero, oscillation begins with a frequency and amplitude regulated by *p* and λ. Y shows the output variable of oscillation (Baghdadi et al., 2019).

Basic assumptions of this model were employed for the functional behavioral model similar to the study by Baghdadi et al. (2019). Structural parameters and variables of the model were considered to match the behavior and nature of the neuronal unit, with reasons behind choosing the Van der Pol oscillator being those mentioned earlier. The model can represent the oscillatory nature of the activity of neuronal units using the variable Y. The parameter λ represents the intrinsic amplitude of the oscillation of the neuronal unit (Baghdadi et al., 2018), and the parameter *p* demonstrates neuronal unit oscillation frequency.

There were two sensory neuronal stream pathways in this study, one for responding to AT and one for PT. An AT neuronal system, similar to the study by Baghdadi et al. (2019), was considered target and non-target, which had a distractor context that is the same with that in a previous study. In our experiment, the target in the SRT task is to press the key, and the non-target is “not press the key;” in the CRT task, the target is to press the correct key regarding stimuli, and the non-target is not to press the non-target key. CRT task performance has a more complex process; however, it was assumed that it would not affect the number of neuronal units, as the number of choice channels (Guthrie et al., 2009) relates to the number of choices, which is the same as that in the SRT task. Consequently, two functional neuronal unit clusters were taken into account, one for target and one for non-target choices.

The situation in the PT task is different, because it is not a discrete task with a specific stimulus time to account for. Nevertheless, certain specifications can help in finding feature candidates. Similar to previous studies (Hill, 2014; Kobler et al., 2018), the size of the target was believed to have been accounted for as a visual sensory feature; however, given the existence of only one target size in the whole sequence, it appears that the effects of easy and hard tracking would be applicable in other performance parameters. Therefore, target size was not taken into account as a separate neuronal unit. In addition, according to the primary assumption regarding vertical tracking, target movement speed can be a feature as well (Kobler et al., 2018; Kieslich et al., 2020). As previously pointed out in the PT experiment section, the issue pertains to significantly rapid changes in constant target speeds, rendering them undetectable by the participants; however, it remains an attention-demanding feature. Subsequently, the average speed was considered constant during the experiment and attention-demanding at all times. Considering this explanation, we assume that the average speed is a separate DT feature. Another feature appears to be movement direction. Since a change in directions solely occurs at the positive and negative peaks of the force function with random amplitudes, it can also be considered as an attention-demanding feature. As a result, there were two separate neuronal units for representing the PT task functional procedure process, which entailed the average speed (S) and change in direction of the positive and negative peaks (D).

**Figure 3:**
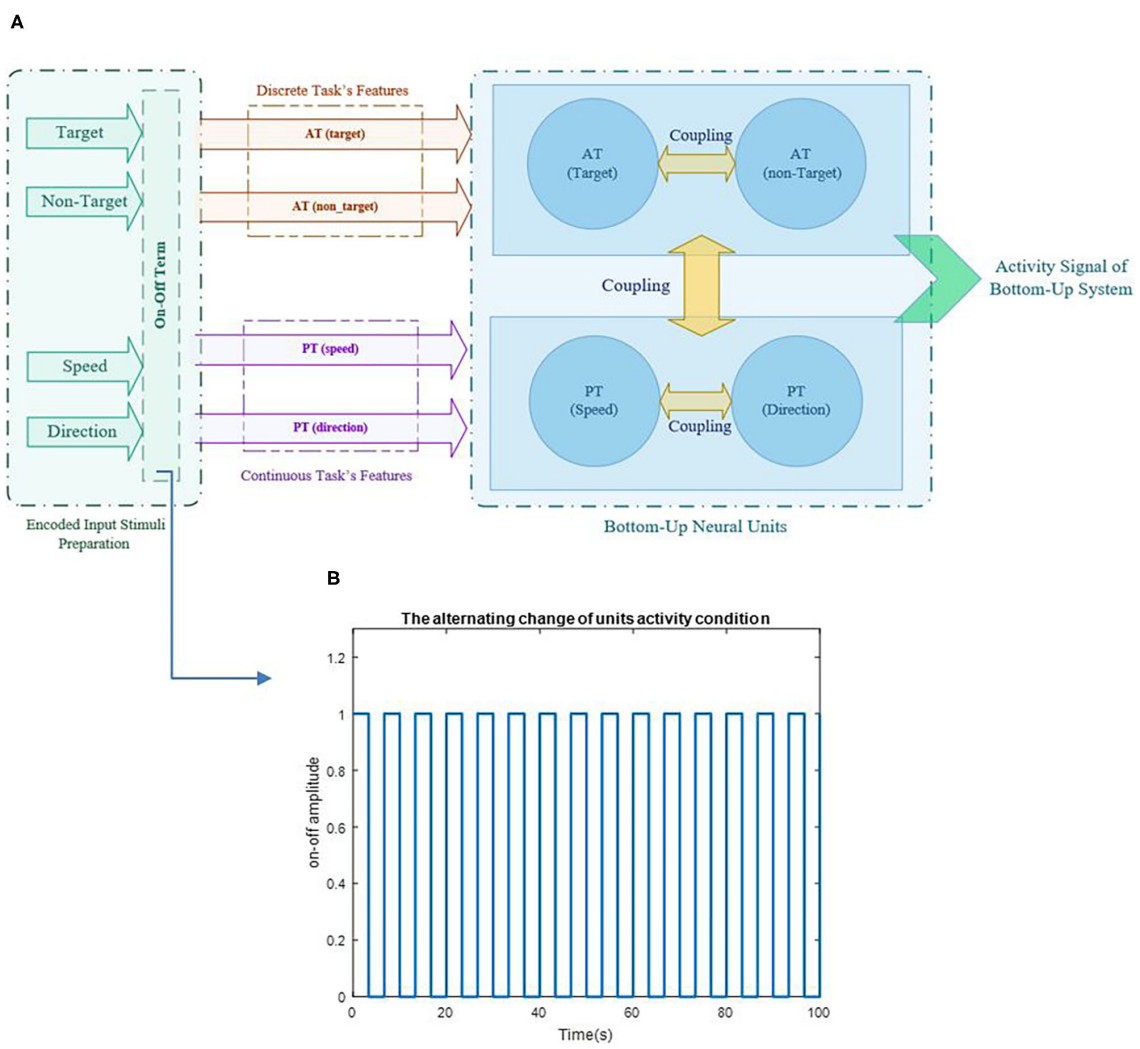
Schematic of the proposed model's structure and illustration of alternative change in unit's activity based on the on-off term are represented in pictures **(A,B)**, respectively. The parameter ***a*** varies for each task in each sequence based on the variability of the task's output and provides a representation of different activities in each task of each sequence.

The bottom-up process is based on sensory inputs and their responses, which are the auditory stimuli in AT. We considered one oscillator for each neuronal unit cluster to model the bottom-up system process. As a result, two Van der pol oscillators were taken into account for the AT process in the bottom-up attention model. These two oscillators are assumed to be sensitive to target and non-target features. In other words, when the target is being decided, the neuronal unit of the related response would represent the highest activity, and the other unit would be in its prior activity. As this study was conducted as DT, it would be practical to consider the interaction between the two PT and AT tasks. The model's structure is depicted in Figure 3A. According to the statistical results, fewer errors happened during higher performances of both tasks in the majority of sequences. Therefore, a connection coefficient exists between AT and PT performances that couple them together. By drawing inspiration from a previous study (Baghdadi et al., 2019) where another oscillator was regarded as the top-down controller system applied to the feature oscillation, only certain relevant coupling phrases were added to the oscillators to enable PT-AT interaction, and a new term was added to state the differences between the sequences. The coupling term is as shown in (2).


(2) $$\begin{array}{l} B_{AT}\left(0.5+\sqrt{a_{AT}.\pi .t}/2\right)(A_{trg}\sin\left(\omega_{trg}\right)+A_{ntrg}\sin\left(\omega_{ntrg}\right))\;\\ +\;B_{PT}\left(0.5+\sqrt{a_{PT}.\pi .t}/2\right)(A_{S}\sin\left(\omega_{S}\right)+\;A_{D}\sin\left(\omega_{D}\right))\; \end{array}$$

where *B*_*AT*_ and *B*_*PT*_ are the coefficients of AT and PT and are considered as “coupling parameters”, and *A*_*trg*_, *A*_*ntrg*_, *A*_*s*_, and *A*_*D*_ represent the coefficients of the target, non-target, S, and D, respectively. Values of the coupling parameters change between 0 and 2, in which 0 is referred to as two independent units, and two determines two fully interacted units (0 < A <2). A = 1 is considered a normal interaction condition of the coupling parameter between two units. These values have been chosen hypothetically and do not have physiological references. The source of the features is applied by sinusoidal reference that ω_trg_, ω_ntrg_, ω_S_, and ω_D_ are defined as the target, non-target, S, and D's unit's frequency, respectively. It has been understood from previous studies that when all tasks are performed simultaneously, they do not use the processor's full capacity (Schubert, 2008; Schubert and Strobach, 2018). According to the bottleneck theory, the second task is delayed until the first task is done (Netick and Klapp, 1994; Pashler et al., 2000; Schubert and Szameitat, 2003). Since all relationships in the brain happen based on neurotransmitters, it is reasonable to assume that this role is executed by one neurotransmitter and, is therefore, limited (Lo and Wang, 2006). Since the activity of the units are dependence to each other, to make the brain resources optimum, we used the additional term to control the activity order of the feature unit's so all the units would not activate at their highest capacity constantly. Each task's process is like a substrate that tries to absorb energy and attention, and the term $\left(0.5\;+\;\sqrt{a.\pi .t}/2\right)$ seems to be proper, because it can make oscillatory resources with on and off conditions and fast switching between the two tasks. This oscillatory alternative term will be addressed as “on-off term.” The on-off term controls the unit's activity and, in some aspects, can be considered as attentional control policy (Cliff, 1973; Thissen et al., 2014; Baghdadi et al., 2019). The on-off term could describe different performances that the units are involved in and characterize the tasks' nature, since each task requires a specific amount of attention (Castellanos et al., 2005; Ghorbanian et al., 2013; Katsuki and Constantinidis, 2013). The *a*_*AT*_and *a*_*PT*_ parameters are related to the variability of the performance, since variability is counted as the attentional diversion indicator in various studies (Voss et al., 2004; Thissen et al., 2014; Loveys et al., 2021). Therefore, the value of *a*_*AT*_and *a*_*PT*_ is estimated based on the scale of the variance of each task in different sequences. The coefficients *a*_*AT*_ and *a*_*PT*_ represent the on-off term parameters $\left(0.5+\sqrt{a.\pi .t}/2\right)$ of AT and PT tasks, respectively. A sample of the alternative change for units' activity based on the term $\left(0.5+\frac{\sqrt{a.\pi .t}}{2}\right)$ is shown in Figure 3B.

According to Figure 3, the activity of the units is assumed to be in a one and zero condition based on the level of attention required for the tasks, i.e., for the most attentional-demanding task, the on-off term would be mostly in the one and rarely in the zero condition. Therefore, saturation on-off diagrams would be defined simply as the constant one value. So, the non-attentional demand task would be looking more like zero all the time. Based on simulations, we are convinced that the on-off term $\left(0.5+\sqrt{a.\pi .t}/2\right)\;$would always be in the “on” condition when the difference between the “on” and the “off” is <0.05 (*a* <0.05). The 0.05 interval acquires *a* = 20, so we consider the saturation value for the *a* parameter in the on-off term to be 20 (0 < *a* <20), and the variability of the tasks in different sequences would be scaled to be between the minimum and the maximum of these limits.

The equations represent the two neuronal units that are sensitive to target and non-target, respectively, Equations 3, 4:


(3) $$\begin{array}{l} {\overset{..}{Y}}_{AT_{trg}}-\left(\lambda_{AT_{trg}}-Y_{AT_{trg}}^{2}\right){\dot{Y}}_{AT_{trg}}+p_{AT_{trg}}^{2}Y_{AT_{trg}}\\ \;=\;A_{trg}\sin\left(\omega_{trg}\right)+A_{ntrg}\sin\left(\omega_{ntrg}\right)+Coupling \end{array}$$

(4) $$\begin{array}{l} {\overset{..}{Y}}_{AT_{ntrg}}-\left(\lambda_{AT_{ntrg}}-Y_{AT_{ntrg}}^{2}\right){\dot{Y}}_{AT_{ntrg}}+p_{AT_{ntrg}}^{2}Y_{AT_{ntrg}}\\ \;=\;A_{trg}\sin\left(\omega_{trg}\right)+A_{ntrg}\sin\left(\omega_{ntrg}\right)+Coupling, \end{array}$$

where the subscripts of *A*_*trg*_and *A*_*ntrg*_ represent the auditory task's target and non-target, respectively. Equations 3, 4, respectively, respond to the target and non-target, which are the options of the task's choice. Stimulation features affect the sensory system by controlling oscillation system frequency as a characteristic of neuronal unit activity (Gabbiani and Metzner, 1999; Koepsell et al., 2010; Watrous and Ekstrom, 2014; Goris et al., 2015). For instance, the sound sample's pitch can be a feature encoded into different activities of neuronal units. Moreover, previous studies have shown that the amplitude of neural activity can also be affected (Röhl and Uppenkamp, 2012). As a result, the target and non-target characteristics represented by different frequencies of oscillatory waves of ω_trg_ and ω_ntrg_ were considered here. *A*_*trg*_ and *A*_*ntrg*_ represent the amplitude and the strength of the target and the non-target, respectively. If the frequency ω was close enough to the oscillator's frequency (*p*), then t stimulus wave could force the Van der Pol oscillator to synchronize. In this study, the effect of the top-down controller was not investigated, as the purpose involved examining the bottom-up effect on different features of DT's components. The terms of resources (right part) of both equations are similar, because we try to consider the units' interaction together. Therefore, the PT and AT units would be involved in all the features *via* synchronization occurrence, and all the units need to interact to make this happen.

Regarding the PT task, two neural units were assumed to be representatives of the S and D features. Subsequently, these two oscillators were assumed to be sensitive to speed and direction change, as in Equations 5, 6, respectively.


(5) $$\begin{array}{l} {\overset{..}{Y}}_{PT_{S}}-\left(\lambda_{PT_{S}}-Y_{PT_{S}}^{2}\right){\dot{Y}}_{PT_{S}}+p_{PT_{S}}^{2}Y_{PT_{S}}\\ \;=\;A_{S}sin\left(\omega_{S}\right)+\;A_{D}sin\left(\omega_{D}\right)+Coupling \end{array}$$

(6) $$\begin{array}{l} {\overset{..}{Y}}_{PT_{D}}-\left(\lambda_{PT_{D}}-Y_{PT_{D}}^{2}\right){\dot{Y}}_{PT_{D}}+\;p_{PT_{D}}^{2}Y_{PT_{D}}\\ \;=\;A_{S}sin\left(\omega_{S}\right)+\;A_{D}sin\left(\omega_{D}\right)+\;Coupling, \end{array}$$

where the subscripts of PT_S_ and PT_D_ are the neural units of S and direction in the PT task, respectively. The direction feature responds to any change in direction on the vertical axis (positive and negative). Each equation is sensitive to its feature changes. Therefore, if the frequency of the oscillator (*p*) was close to each frequency of the features' frequency (ω_S_ or ω_D_), that unit would involve maximum oscillation with input sensory wave.

According to neuropsychological studies, the activity of a unit can be detectable in the next layer when it reaches a specific threshold (Kurten, 1988; Izhikevich, 2001; Lo and Wang, 2006). This threshold appears to be changeable depending on the nature of the brain's pathway or the accuracy-speed trade-off of the task's performance (Lo and Wang, 2006). In theory, the threshold of activating a unit is assumed to be 1, since this threshold can have relationships with dopamine arising from active units (Guthrie et al., 2009), but for simulation's sake, with a slight change, we assumed the threshold to be 1.1 to get more acceptable results.

Sampling time of the entire modeling simulation was set to 0.01 s, and the initial condition was determined as Y_trg_ (0) = Y_ntrg_ (0) = Y_S_ (0) = Y_T_ (0) = 0.5, since we assumed all neural units to act at a medium level before any task starts (Ghorbanian et al., 2015; Baghdadi et al., 2018). In addition, the bifurcation parameter λ was set to 0.2. Since oscillations require the value of λ to be greater than 0, it is considered as the oscillation amplitude. The λ's value should also be lower than 1, because the stimuli (for both AT and PT) are coded to be a simple wave with certain frequency and amplitude. If λ was greater than 1, there would be no frequencies, and the model would become more problematically complex. Any change in initial values may also lead to uncontrollable conditions, as they depend on the previous state of the units. Uncontrollable condition refers to any condition that makes oscillator decay zero and not oscillate or cause chaos. Therefore, the arbitrary parameters should be fixated within an acceptable range to be associated with the definition of force synchronization and be significant for task indices. In AT, considering the study by Baghdadi et al. (2019), the target and non-target were represented by ω_trg_ = 6 Hz and ω_ntrg_ = 10 Hz, respectively.

Additionally, prior research shows that PT contains information about the target and direction coded in a low-frequency EEG signal while activating the parietal area (Kobler et al., 2018; Hung et al., 2020). Therefore, we considered two lower frequency values for the features of PT as ω_S_ = 3.5 Hz and ω_D_ = 4.5, and were used regarding the S and D features in PT. These frequencies were decided to be closer together (3.5 and 4.5 Hz) in comparison to target and non-target features (6 and 10 Hz), because the S and D features are both desired and depend on each other. In other words, when one cannot change direction in the proper moment, it probably would also lose the speed pursuit. Besides, we choose the target frequency closer to the S and D feature instead of the non-target (4.5 <6 < <10), since acceptable performance is mainly defined under the three desired features, S, D, and target, and based on results of the simulation, more similarity in frequencies makes stronger bound between the units and produces higher activity in output. Notably, every AT stimulus occurs at pseudo-random time intervals in the range of 10–15 s; however, for the continuous task (PT), there was no interval time or very small intervals for features that entail attention-demanding factors. Consequently, it appears that their frequency should be higher than the input wave of the AT features. Alternatively, there is a significant difference between target and non-target in AT, as the target neural unit is desired. In turn, this would cause a discriminative difference between their activity frequencies. In PT, however, both features are desired together, i.e., attention should be spent on both speed and direction. Consequently, their frequencies should not vary considerably. As mentioned in the simulations section, the higher similarity between S and D features leads to more effective activities in both neural units. Nonetheless, increasing signal strengths can work both ways for target and non-target units.

### Results

In this section, the results of the experiment and the model's simulations are presented.

#### Pre-Statistical Analysis

We interpreted the results by conducting repeated measured ANOVA tests on RT and H. However, before conducting the ANOVA, general assumptions for the analysis of variance were checked. These general assumptions included the continuous variables, normal distribution, and homogeneity of variance for the dependent variables (Ghasemi and Zahediasl, 2012). Independence of the variables is assumed, because each participant is experimenting thoroughly independent from the others. Since the subject's number is 25, we can assume that the variables are normally distributed; however, the Kolmogorov-Smirnov test in SPSS confirms that the assumption of normality is not rejected (*p* > 0.05). The homogeneity of Levene's equality test of errors in variance shows that the errors in variance are equal across the groups (*p* > 0.1). Also, the result of Mauchly's test on RT and H shows that the assumption of sphericity is not violated (*p* > 0.05).

#### Experiment Results

Mean RTs and error rates of AT k and hesitation, and the error rate of PT are presented in Table 2.

**Table 2:** Mean response times, error rates, and hesitation rates in the experimental conditions.

**Overall statistics**								
**Seq (AT, PT)**	**Time-based features**				**Error-based features**			
	**RTs:** **Time of response made - stimulus time** ***(ms)***		**H rates** **(% ratio to sequence=** hesitated duration / sequence duration*100)		**AT error rates** **(% Wrong answers=** **Correct answers / whole stimuli** *100)		**PT error rates** **(% Cursor out of target=** duration of cursor inside the target/ whole sequence*100)	
	**SRT**	**CRT**	**EPT**	**HPT**	**SRT**	**CRT**	**EPT**	**HPT**
**Single tasks**								
Seq (1,0)	330 (± 7)				0.29 (± 0.01)			
Seq (2,0)		550 (± 11)				8.86 (± 0.08)		
Seq (0,1)			4.16 (± 0.36)				0.09 (± 0.06)	
Seq (0,2)				4.5 (± 0.38)				0.21 (± 0.011)
**Dual tasks**								
Seq (1,1)	329 (± 7)		5.08 (± 0.34)		0.57 (± 0.03)		0.1 (± 0.07)	
Seq (1,2)	357 (± 8)			5.24 (± 0.41)	0.29 (± 0.01)			0.26 (± 0.14)
Seq (2,1)		593 (± 12)				9.43 (± 0.08)	0.05 (± 0.04)	
Seq (2,2)		629 (± 13)		7.7 (± 0.52)		4.86 (± 0.06)		0.24 (± 0.16)

*Seq, sequence; RT, response time; H, hesitation; ms, milliseconds*.

*For more information on the sequences, see Table 1. The values in brackets represent mean standard errors*.

The results of a repeated measures ANOVA on ATs (SRT vs. CRT) and PTs (EPT vs. HPT) under both ST and DT conditions showed that the participants had more AT errors in the CRT task than in in SRT [*F*_(1, 24)_ = 45.23, *p* < 0.001, $n_{\text{p}}^{2}$ = 0.65], and more AT errors in HPT than in EPT tasks [*F*_(2, 19)_ = 4.73, *p* = 0.013, $n_{\text{p}}^{2}$ = 0.17]. Given the interaction between AT and PT, the AT error rate between SRT and CRT was larger in HPT compared to the EPT task [*F*_(2, 19)_ = 3.93, *p* = 0.026, $n_{\text{p}}^{2}$ = 0.14]. RTs associated with correct responses were larger for CRT than for the SRT task [*F*_(1, 24)_ = 232.8, *p* < 0.001, $n_{\text{p}}^{2}$ = 0.91] and larger for HPT than for EPT [*F*_(2, 48)_ = 12.75, *p* < 0.001, $n_{\text{p}}^{2}$ = 0.35]. Similarly, the interaction between AT and PT [*F*_(2, 48)_ = 6.34, *p* = 0.004, $n_{\text{p}}^{2}$ = 0.21] confirmed a stronger RT difference between HPT and EPT tasks in the CRT task than in SRT. Regardless of the AT error rate, the PT error rate exclusively showed relevance of the results to PTs. In other words, the participants made more PT errors in HPT than in EPT [*F*_(2, 46)_ = 58.96, *p* < 0.001, $n_{\text{p}}^{2}$ = 0.72]. According to the analysis, the H factor demonstrates larger pauses in CRT than in SRT in DTs [*F*_(2, 44)_ = 9.08, *p* < 0.001, $n_{\text{p}}^{2}$ = 0.29], and no significant differences were found regarding H factor results in HPT and EPT [*F*_(1, 22)_ = 3.18, *p* = 0.088, $n_{\text{p}}^{2}$ = 0.13]. An interaction of AT and PT tasks showed a greater difference in H rates between CRT and SRT in HPT than in EPT.

These results support the general idea that HPT is more challenging than EPT in both ATs (CRT and SRT). It also indicates a speed-accuracy trade-off between the CRT and HPT tasks, both of which are recognized as hard tasks in their respective categories. In addition, the PT errors and H findings indicate that raising the level of difficulty is more effective in AT performance than PT performance.

#### Simulation Results

In this section, the simulation results are presented as the push of the activities of each neural unit defined earlier. According to Baghdadi et al. (2018), push of the signal is the pattern of peaks of sinusoidal activity that a signal alters, which is reasonable, since it shows the highest level of activity change. Here, push of the activity signals was employed without losing the content of the neuronal unit's activity to offer more clarity and easier traceability. It should be noted that the simulations are considered for one stimulus, and that the units of ω are considered to be in Hz, but for the sake of brevity, the unit is not mentioned in captions of the Figures. Besides, the x-axis represents time, but we did not mention the unit of time as in Baghdadi et al. (2018) since we are investigating the behavior in the output, and comparisons are made on the output's pattern with recorded behavioral data (second) and EEG signals (millisecond) that include different time units. This would not lose any content, since our purpose is to investigate change in the behavior of the signals.

The results are obtained for one general stimulus of a discrete task but not a specified event of PT. Therefore, parameters based on these feature frequencies apply the determined frequency of speed and direction change for PT, and, the general results are not obtained from specific speed or direction change during a sequence.

Since we would compare the simulation results with other research studies, including the EEG signals in the prediction section, it is acceptable to use the RMS^4^ value as a measure of comparing simulated results together. The RMS value of the signal is known as quadratic means, which would consider the alternation effects of sinusoidal curves. Regarding that, in the proposed model, more alternations can be interpreted as higher activity of the related unit. A higher RMS value can be interpreted as the reaction of parameters to the unit's activity (Altahat et al., 2012). To reach this aim, we decomposed the output signal into 3–8 (depending on the complexity of signal) sine harmonies, and then we calculated the RMS value. Due to brevity, we only report the result of the RMS value and the variability of signals.

#### Simulation Results of Experiment Sequences

**Figure 4:**
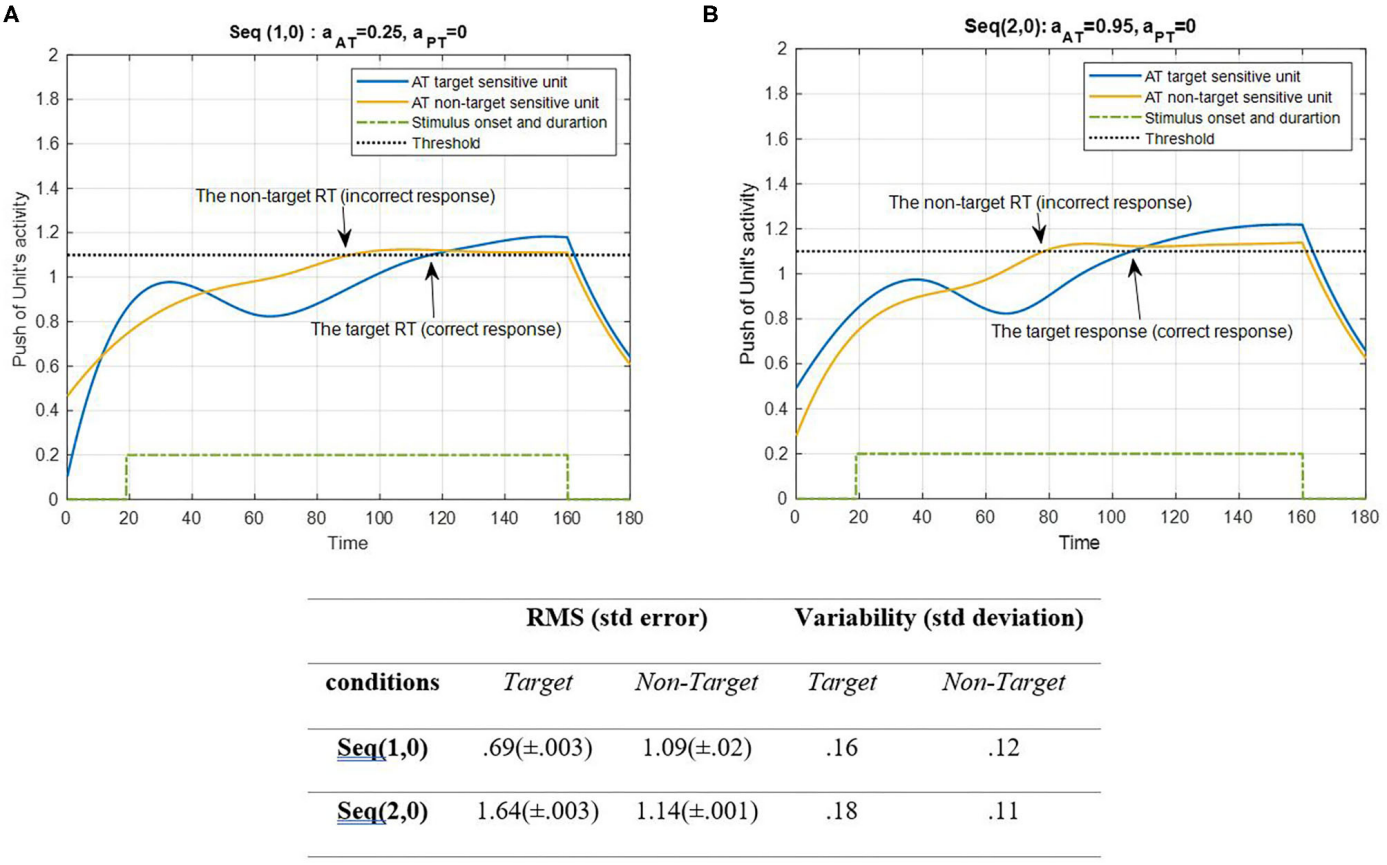
Activity of AT's target and non-target units for seqs (1, 0) and (2, 0) is shown in **(A,B)**, respectively, and the RMS value and variability of the signals are shown in the table. The sequences are considered under ST conditions and do not include the activity of PT units (A_S_ = A_D_ = 0). The tasks parameters are estimated from the variability of observations (RT and H) and errors: a_AT_ = 0.25; a_PT_ = 0 for seq (1, 0) and a_AT_ = 0.95; a_PT_ = 0 for seq (2, 0). The blue curve represents the activity of the neuronal unit oscillator sensitive to the AT's target (Equation 3). The yellow curve represents the activity of the neuronal unit oscillator sensitive to the AT's non-target (Equation 4). The green dashed curve demonstrates the stimuli's onset and duration. The black dashed line shows the effective threshold that activates the related unit [λ_trg_ = λ_ntrg_ = 0.2; A_trg_ = A_ntrg_ = 1; B_PT_ = 0; B_AT_ = 1; Y_trg_(0) = Y_ntrg_(0) = 0.5; p_trg_ = 6; p_ntrg_ = 10; ω_trg_= 6; ω_ntrg_= 10].

The results of task sequences are presented here. Each sequence contains all common parameters along with *a*_*AT*_ and *a*_*PT*_, the parameter in the on-off term that separates each task in every sequence. Here, the result of AT under ST conditions (SRT and CRT) is shown in Figure 4.

**Figure 5:**
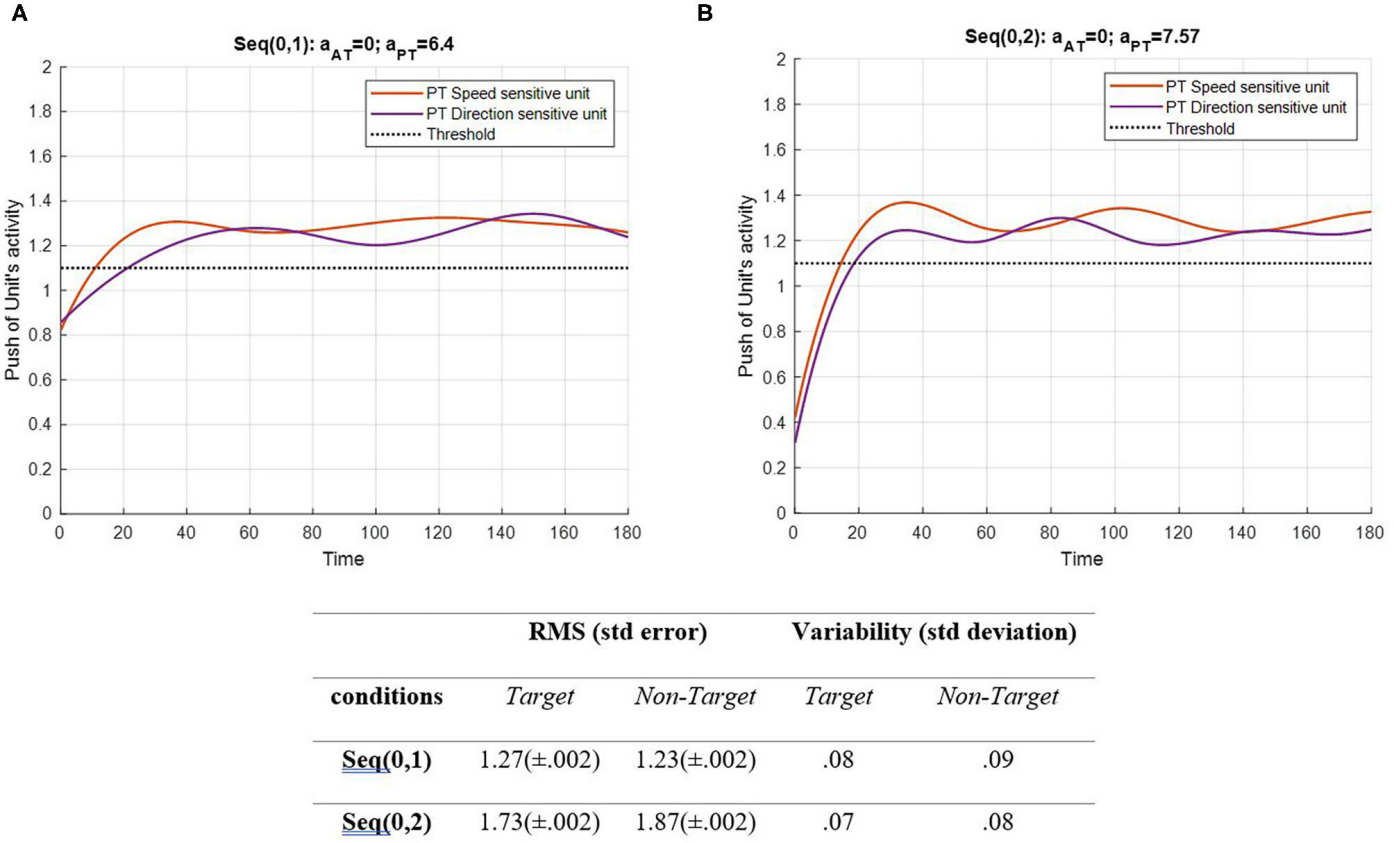
Activity of the PT's speed and direction units for seqs (0, 1) and (0, 2) is shown in **(A,B)**, respectively, and the RMS value and variability of the signals are shown in the table. The sequences are considered to be under ST conditions and do not include the activity of AT units (A_trg_ = A_ntrg_= 0). The tasks' parameters are estimated from the variability of observations (RT and H) and errors: a_AT_ = 0; a_PT_ = 6.4 for seq (0, 1) and a_AT_ = 0; a_PT_ = 7.57 for seq (0, 2). The red curve represents the activity of the neural unit oscillator sensitive to the PT's speed (Equation 5). The purple curve represents the activity of the neuronal unit oscillator sensitive to the PT's direction (Equation 6). The black dashed line shows the effective threshold that activates the related unit [λ_S_ = λ_D_ = 0.2; A_S_ = A_D_ = 1; B_PT_ = 1; B_AT_ = 0; Y_S_ (0) = Y_D_ (0) = 0.5; *p*_S_ = 3.5; *p*_D_ = 4.5; ω_S_ = 3.5; ω_D_ = 4.5].

Figure 4 represents the simulation of target and non-target ATs. The RMS value of the target and non-target features of seqs (1, 0) and (2, 0) shows that the target feature in the CRT single task is activated more than the other units and the non-target feature in the SRT single task. The unit of non-target is more activated than the target unit to avoid an unwanted response. This may be because of the simplicity of the target of the SRT task. However, the variability of both conditions shows more alternations in target units than the non-target. There is a stimuli period during which the oscillator would be activated, similar to the real AT. Following the period of stimuli, the signal would decrease to baseline level. According to Figure 4, the push activity of both AT's target and non-target units passed the activation threshold after a period of desynchronization. To avoid complications, we decided not to consider the top-down controller in this study; subsequently, the non-target can be activated in bottom-up system simulation. Next, the results of PT features under ST conditions are represented in Figure 5.

**Figure 6:**
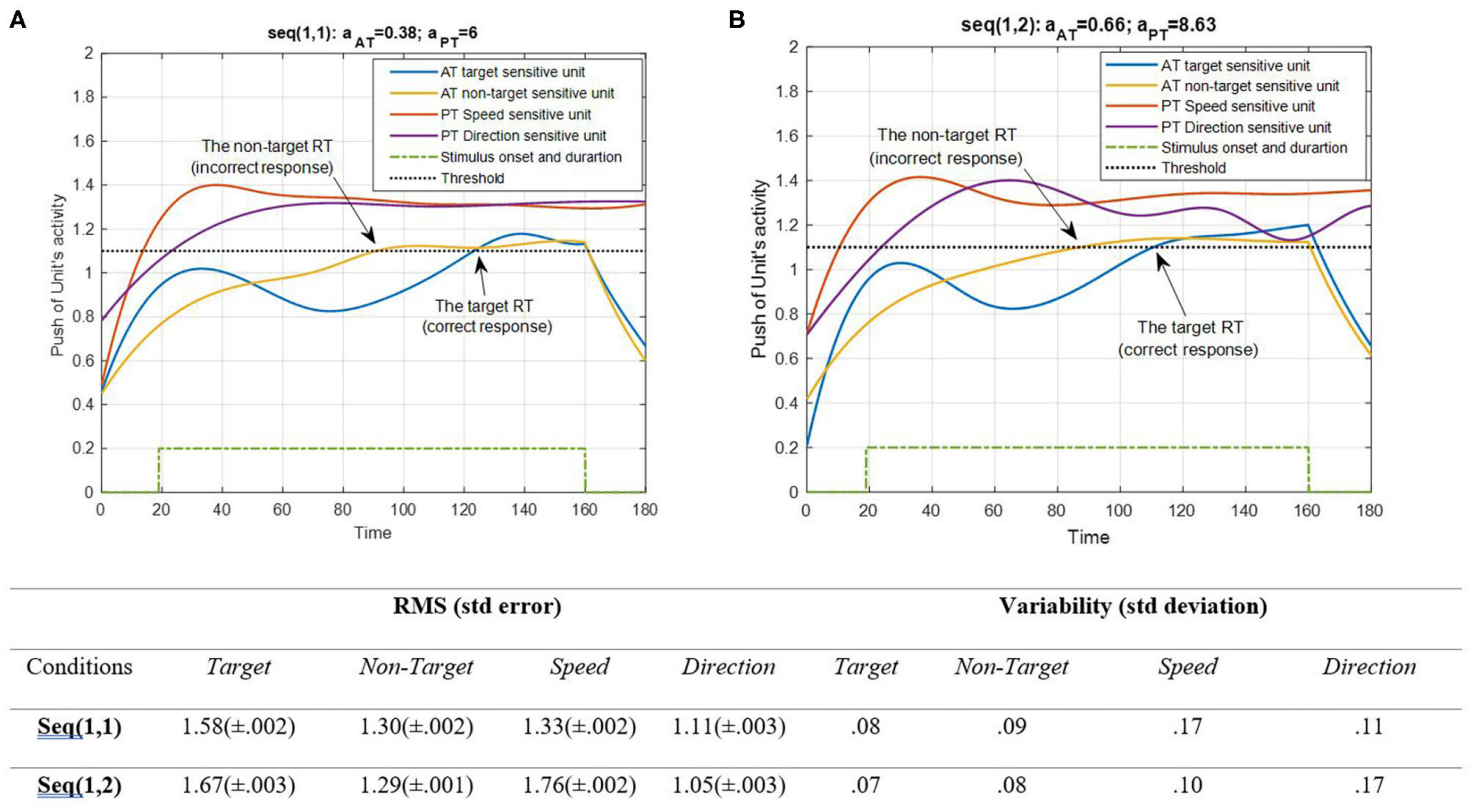
Activity of AT and PT's target, non-target, speed, and direction units for seqs (1, 1) and (1, 2) is shown in **(A,B)**, respectively, and the RMS value and variability of the signals are shown in the table. The sequences are considered under DT conditions (A_trg_ = A_ntrg_ = A_S_ = A_D_ = 1). The tasks' parameters are estimated from the variability of observations (RT and H) and errors: a_AT_ = 0.38; a_PT_ = 6 for seq (1, 1) and a_AT_ = 0.66; a_PT_ = 8.63 for seq (1, 2). The blue and yellow curves represent the activity of the neuronal unit oscillator sensitive to the AT's target (Equation 3) and non-target (Equation 4), respectively. The red and purple curves represent the activity of the neuronal unit oscillator sensitive to the PT's speed (Equation 5) and direction (Equation 6). The green dashed curve shows the AT stimuli's onset and duration. The arrows show the time of making a response in AT. The black dashed line demonstrates the effective threshold that activates the related unit [λ_trg_ = λ_ntrg_ = λ_S_ = λ_D_ = 0.2; A_trg_ = A_ntrg_ = A_S_ = A_D_ = 1; B_PT_ = B_AT_ = 1; Y_trg_(0) = Y_ntrg_(0) = Y_S_ (0) = Y_D_ (0) = 0.5; p_ntrg_ = 10; p_trg_ = 6; p_D_ = 4.5; p_S_ = 3.5; ω_ntrg_ = 10; ω_trg_ = 6; ω_D_ = 4.5; ω_S_ = 3.5].

**Figure 7:**
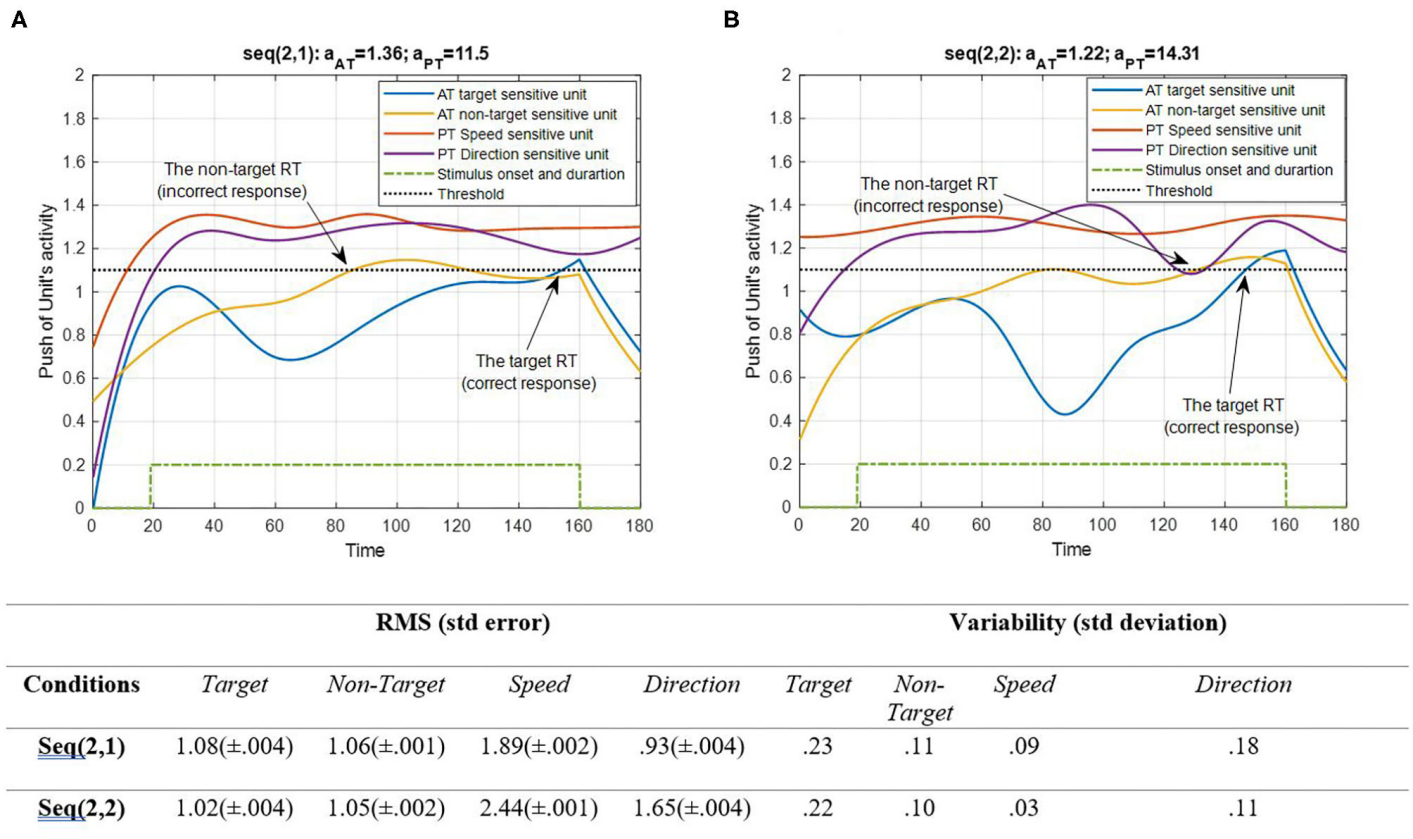
Activity of AT and PT's target, non-target, speed, and direction units for seqs (2, 1) and (2, 2) is shown in **(A,B)**, respectively, and the RMS value and variability of the signals are shown in the table. The sequences are considered under DT conditions (A_trg_ = A_ntrg_ = A_S_ = A_D_ = 1). The tasks' parameters are estimated from the variability of observations (RT and H) and errors: a_AT_ = 1.36; a_PT_ = 11.5 for seq (2, 1) and a_AT_ = 1.22; a_PT_ = 14.31 for seq (2, 2). The blue and yellow curves represent the activity of the neuronal unit oscillator sensitive to the AT's target (Equation 3) and non-target (Equation 4). The red and purple curves represent the activity of the neuronal unit oscillator sensitive to the PT's speed (Equation 5) and direction (Equation 6). The green dashed curve shows the AT stimuli's onset and duration. The arrows show the time of making a response in AT. The black dashed line demonstrates the effective threshold that activates the related unit [λ_trg_ = λ_ntrg_ = λ_S_ = λ_D_ = 0.2; A_trg_ = A_ntrg_ = A_S_ = A_D_ = 1; B_PT_ = B_AT_ = 1; Y_trg_(0) = Y_ntrg_(0) = Y_S_ (0) = Y_D_ (0) = 0.5; p_ntrg_ = 10; p_trg_ = 6; p_D_ = 4.5; p_S_ = 3.5; ω_ntrg_ = 10; ω_trg_ = 6; ω_D_ = 4.5; ω_S_ = 3.5].

Figure 5 shows that PT feature-related neuronal units are activated following a desynchronization period and remain activated during the procedure as expected. Notably, the difference between the RMS values of each unit describes the similarity between the features. In the EPT in seq (0, 1), the RMS value represents lower activity than in seq (0, 2). The HPT task requires more attention to perform, which leads to higher activity of the neural units. It is expected to have higher values and alternations in seq (0, 2). Also, the variability of both features in the two conditions shows a slight change in the patterns. As mentioned earlier, the frequency of non-target was assumed to have least similarities with target, S, and D frequencies. In addition, the frequencies of S and D are assumed to be more similar, as both are the desired activating units. Under DT conditions, this similarity gains more importance, as illustrated in Figures 6, 7. The result of AT and PT under DT conditions (SRT-EPT and SRT-HPT) is shown in Figure 6.

According to Figure 6, the desired features of the target, S, and D, which are acceptable in terms of attention demand and energy resource, are superior to the effectiveness threshold. The non-target has also reached the threshold in BU. The RMS value shows that under difficult PT conditions, the amplitude of the target and speed feature units increases more (0.09 and 0.43 increments, respectively). However, the variability of signals shifts from speed to direction in the HPT condition with a slight change in target and non-target features. The coupling parameter added to the equations is responsible for the increase in activity. This is because the coupling parameter generates a strong interaction between the similar frequencies of the features, given the fact that no top-down attentional control was applied to the system. Subsequently, as the frequencies of the desired features were intended to be closer together, this can be considered a reliable result. The result of AT and PT under DT conditions (CRT-EPT and CRT-HPT) is shown in Figure 7.

According to Figure 7, unit synchronization occurs with more complications in CRT than in SRT (Figure 6), and this leads to more delay in passing the threshold, which explains the increased RT in CRT compared with SRT. The RMS value shows more activity of neural units in speed and direction features in the HPT condition of the CRT task. Also, the variability of signals shows more variations of target and direction features. The RT of the model activity according to the Figures was calculated as the time that the push of activity reaches the threshold, and the difference of target hit time with stimulus time is considered as the RT of AT, as in (Equation 7).


(7) $$\begin{array}{r} RT=T_{\text{Target pass the threshold}}-T_{\text{stimulus}}. \end{array}$$

It should be noted that this is different from the RT usually defined in the area of behavioral research, which is the difference between response time and the end of stimulus duration and response time. However, here, hitting time is not quite the response representing time, but it is the time that starts the comprehension of a stimulus, and then the response-making process begins. Therefore, the RT here means the time for the stimulus to be understood and the related unit to start activating. Moreover, the RT of simulation had an 84% correlation with the RT of behavioral observations. We also calculated the variability of the push of S and D featured activity to see if it is related to hesitation. We suppose that this variability has a high correlation with hesitation in the data recorded. In that case, we can conclude that hesitation happens in the stimulus-based response, and that the correlation was 0.26. Hence, the stimulus comprehension of bottom-up attention has low dependency on the hesitation that appears in the experiment.

In all conditions of ST and DT, the non-target feature responses in AT happened earlier than the target responses, which is consistent with observations and other studies. Fast responses (decreased RTs) made after stimulus appearance included more incorrect responses than slow rated responses (increased RTs). This is because there is a balance or trade-off exists between making the fastest response or correct responses which is possible to make in human performance (Pashler, 1994; Kiesel et al., 2010).

#### Simulation of Predicted Results

In this section, EEG studies related to the experiment will be investigated by different manipulations of models' parameters: the coupling parameters, strength factor, and similarity factor. Here, we assumed that the on-off parameter has the same value (a = 1.8) for all sequences to make more generality in the simulation. The purpose of this section is to investigate the generality of the model for any discrete-continuous DT condition under the prediction label. We will explain the simulated results in qualitative methods, and the comparisons between related studies and our simulated outputs and the interpretation of alternations in the patterns. We suggest that this will reveal more strength and validity of the model.

##### The Coupling Parameters

**Figure 8:**
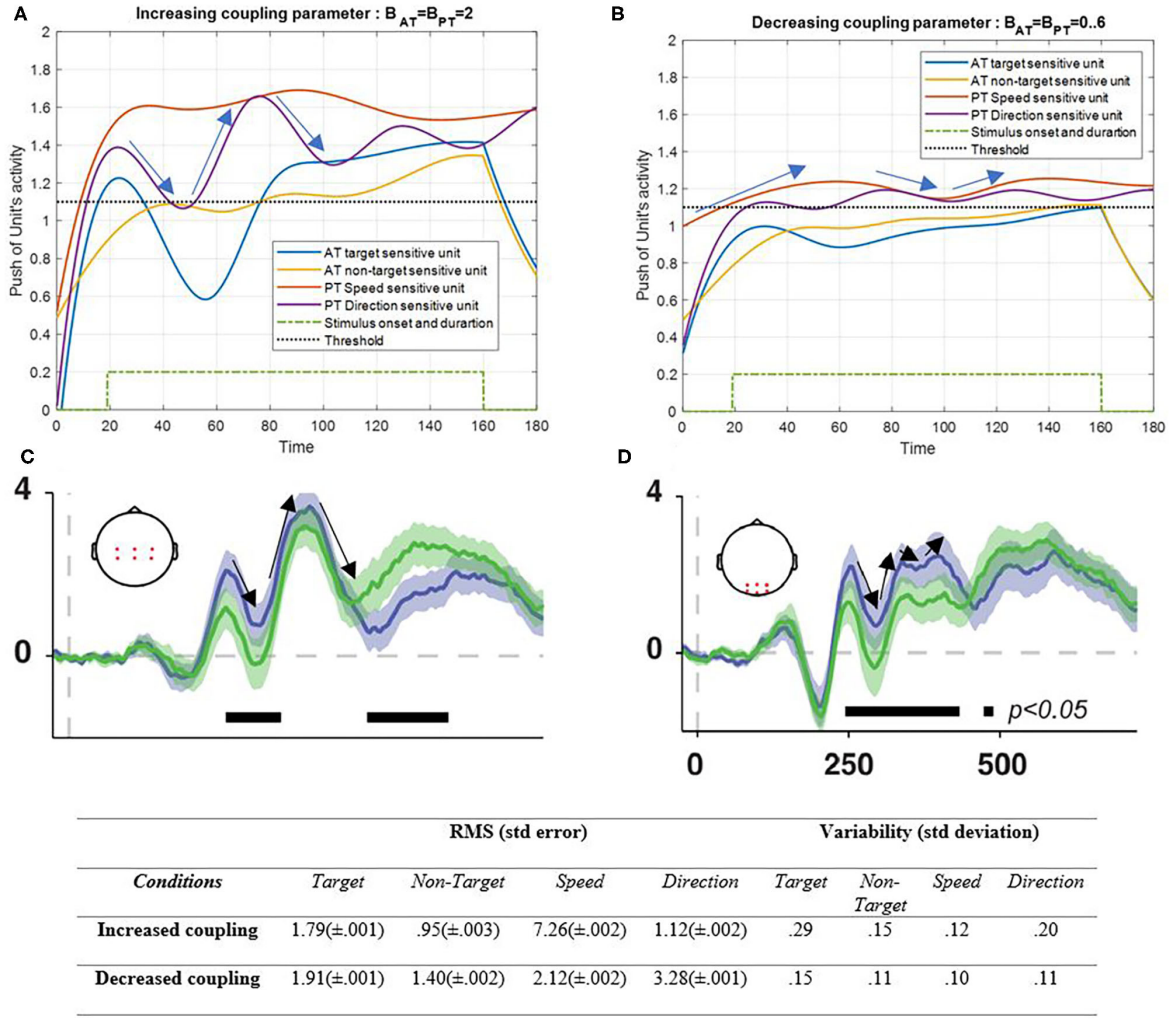
Activity of AT and PT's target, non-target, speed, and direction units, considered under DT conditions (A_trg_ = A_ntrg_ = A_S_ = A_D_ = 1) with increased coupling parameter of both tasks (B_PT_ = B_AT_ = 2) is shown in **(A)** and decreased coupling parameter of both tasks (B_PT_ = B_AT_ = 0.3) is shown in **(B)**. The RMS value and variability of the signals are shown in the table. **(C,D)** show broadband responses to task-relevant (green signal) and irrelevant (purple signal) stimulus onsets [adapted from Myers et al. (2014)]. The blue and yellow curves represent the activity of the neuronal unit oscillator sensitive to the AT's target (Equation 3) and non-target (Equation 4). The red and purple curves represent the activity of the neuronal unit oscillator sensitive to the PT's speed (Equation 5) and direction (Equation 6). The green dashed curve shows the AT stimuli's onset and duration. Finally, the black dashed line shows the effective threshold that activates the related unit [λ_trg_ = λ_ntrg_ = λ_S_ = λ_D_ = 0.2; A_trg_ = A_ntrg_ = A_S_ = A_D_ = 1; B_PT_ = B_AT_ = 2 in the left and B_PT_ = B_AT_ = 0.6 in right pictures; Y_trg_ (0) = Y_ntrg_ (0) = Y_S_ (0) = Y_D_ (0) = 0.5; *p*_ntrg_ = 10; *p*_trg_ = 6; *p*_D_ = 4.5; *p*_S_ = 3.5; ω_ntrg_ = 10; ω_trg_ = 6; ω_D_ = 4.5; ω_S_ = 3.5].

Figure 8 shows the effectiveness of the coupling parameters.

As can be seen in Figure 8A, increasing the coupling parameter (B_AT_ = B_PT_ = 2) of the sensory inputs as a result of synchronization would increase the activity of the desired features. Here, the undesired feature also increases gradually, which can easily be controlled using the top-down attentional control system (Baghdadi et al., 2019). Decreasing coupling parameters led to decrement in activity results that are expected in abnormal situations of a neural interaction (Figure 8). Figures 8C,D represent the average ERPs^5^ of the central and posterior areas of the brain during a working memory task in the presence of target and non-target (distractor) stimuli (Myers et al., 2014). The pattern of the ERP in the posterior area (Figure 8D) has more resemblance to the low coupling condition in the results (Figure 8B). On the other hand, when the coupling increases (Figure 8C), the pattern that matches the central ERP appears (Figure 8A). Also, the RMS value shows that the increasing coupling parameter activates the S feature more than the other features (5.2 difference), which has the most frequent alternation and is a more attentional demand feature, but lack of coupling factor leads to ineffective reactions of all solo units. The variability also shows more alternations in increased coupling parameters.

As will be explained in the discussion section, the effect of lowering the coupling parameter, as shown in Figure 8, seems to be the interaction results in neurodegenerative diseases like Parkinson's disease (Flowers and Robertson, 1995; Talarposhti et al., 2013). On the other hand, as shown in Figure 8, decreasing coupling parameters decreases the activity of desired features because of decreasing synchronization of neuronal units. In this situation, the desired features can barely cross the threshold. This situation can be referred to as low neuronal unit activity, which may result from various impairments. Abnormal interaction of neuronal units of the frontal area in Parkinson's disease is shown to cause abnormality in the frontal area's functionality (Rowe et al., 2002). Since the coupling parameter is the interaction parameter among the units, any decrement is expected to cause dysfunctionality in the output behavior, which is associated with neurodegenerative diseases like Parkinson's disease (Mishra and Thrasher, 2021).

##### Strength Factors

**Figure 9:**
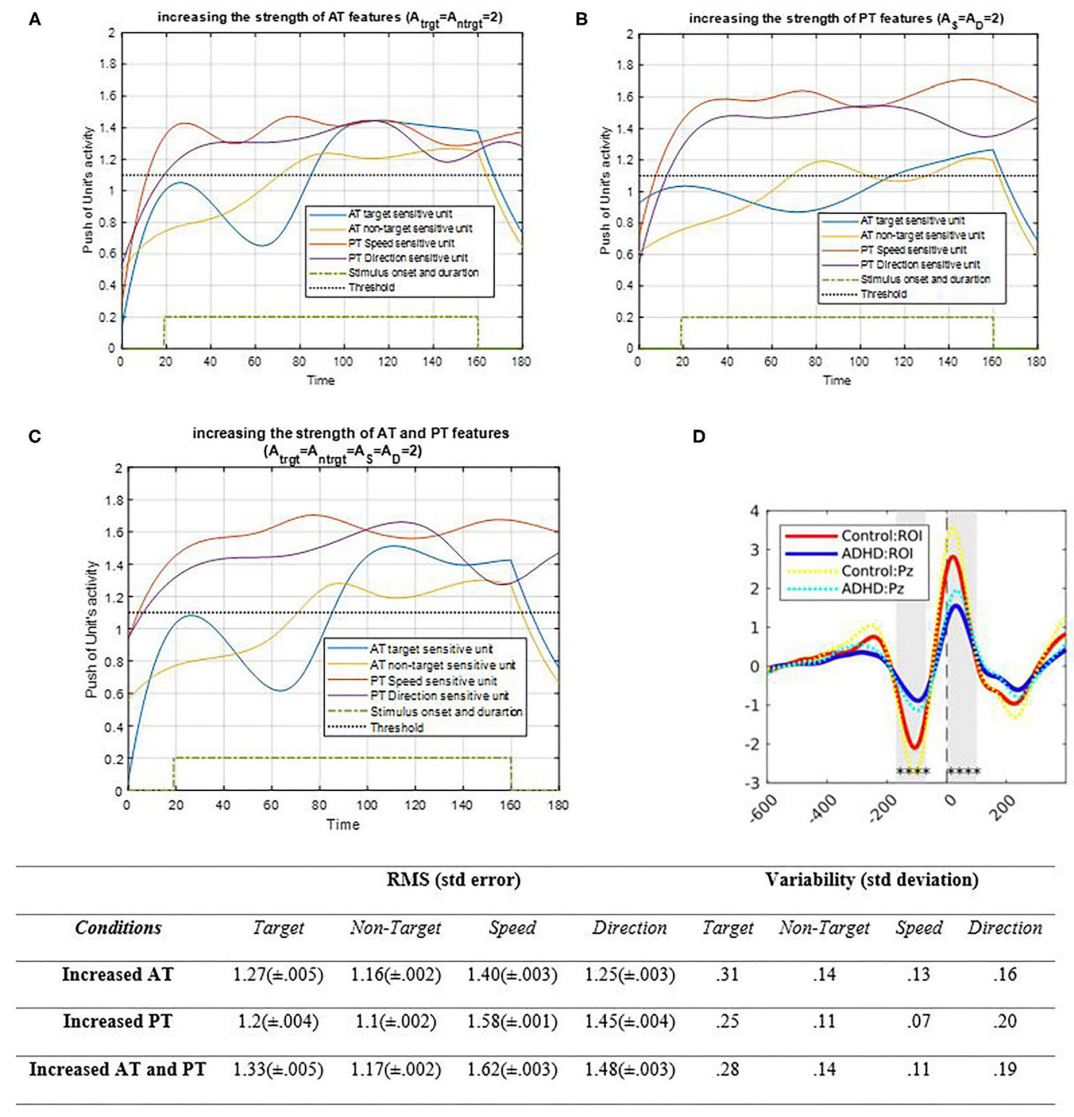
Activity of AT and PT's target, non-target, S, and D units considered under DT conditions with increased strength of AT features is shown in **(A)** (A_trg_ = A_ntrg_ = 2; A_S_ = A_D_ = 1); increased strength of PT features is shown in **(B)** (A_trg_ = A_ntrg_ = 1; A_S_ = A_D_ = 2), and increased strength of both AT and PT features is shown in **(C)** (A_trg_ = A_ntrg_ = A_S_ = A_D_ = 2). The RMS value and variability of the signals are shown in table. **(D)** shows the ERP of the parieto-occipital region during correct trials for control and ADHD groups [adapted from Cowley et al. (2020)]. The blue and yellow curves represent the activity of the neuronal unit oscillator sensitive to AT's target (Equation 3) and non-target (Equation 4). The red and purple curves represent the activity of the neuronal unit oscillator sensitive to the PT's speed (Equation 5) and direction (Equation 6). The green dashed curve shows the AT stimuli's onset and duration. The black dashed line demonstrates the effective threshold that activates the related unit [λ_trg_ = λ_ntrg_ = λ_S_ = λ_D_ = 0.2; B_PT_ = B_AT_ = 1; Y_trg_ (0) = Y_ntrg_ (0) = Y_S_ (0) = Y_D_ (0) = 0.5; *p*_ntrg_ = 10; *p*_trg_ = 6; *p*_D_ = 4.5; *p*_S_ = 3.5; ω_ntrg_ = 10; ω_trg_ = 6; ω_D_ = 4.5; ω_S_ = 3.5].

In addition to the coupling parameters, there are other controlling factors; one of them is strength of the input signal that holds the related sensory input in the signal response stage. The strength of AT and PT features is examined in Figure 9 separately and increases the strength factors.

As shown in Figure 9A, raising the strength of AT's features by increasing the oscillator amplitude increases the activation of related feature units compared to the past. Moreover, since the coupling factor is not zero, the features of PTs are increased slightly. Figure 9B shows how the PT's activity results would change by increasing the strength of the associated oscillator. As expected, the increase in PT's oscillator amplitude increased the activity of the features' neuronal units. Similar to the results obtained from increasing the AT amplitude, raising the PT's feature oscillator amplitude would affect the activities of the AT target and non-target units and enhance them as well. However, since the target frequency is closer to the PT's feature unit's frequencies, it affects the target's units more than those of the non-target. Figure 9C demonstrates that increasing the strength of four oscillators increases the activity of all units; accordingly, considering the coupling parameter, such increase is mostly due to synchronization. Figure 9D shows the ERP of the parieto-occipital area in correct response trials on healthy patients and patients with ADHD. The synchronization decreases in under ADHD condition, as what happens in the lower strength factor of simulation results. Also, the RMS value shows higher increment in features' amplitude in both strengths, increasing compared to increasing AT and PT's strength separately. Also, the variability of target and direction is higher when the strength of features is increased.

##### Similarity Factors

**Figure 10:**
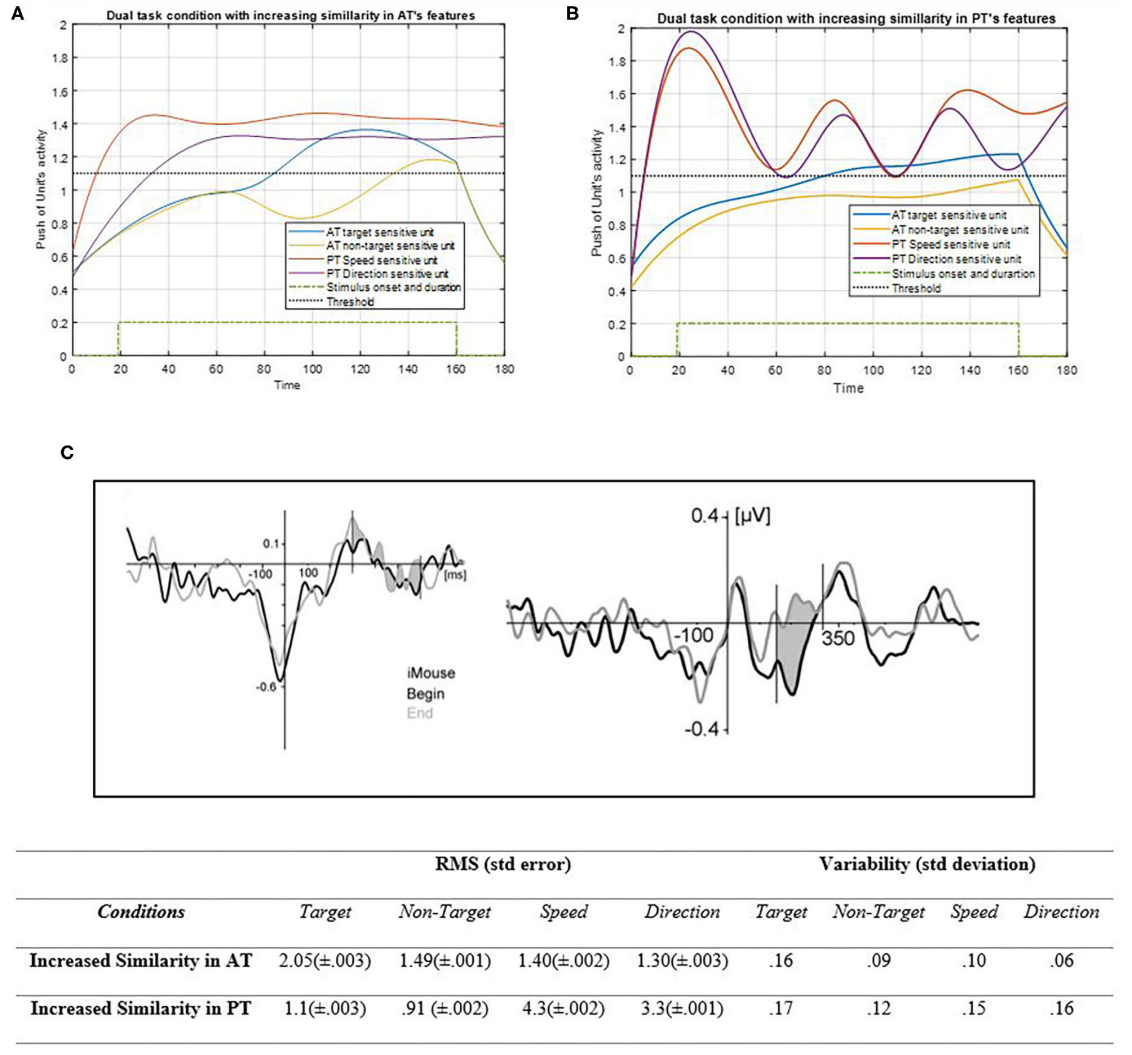
Activity of AT and PT's target, non-target, speed, and direction units considered under DT conditions (A_trg_ = A_ntrg_ = A_S_ = A_D_ = 1) with increasing of the similarity between AT task's features shown in **(A)** (p_ntrg_ = 8; ω_ntrg_ = 8; p_trg_ = 7; ω_trg_ =7) and increased similarity between PT's features shown in **(B)** (p_D_ = 4.1; ω_D_ = 4.1; p_S_ = 3.6; ω_S_ = 3.6). The RMS value and variability of the signals are shown in the table. **(C)** Shows the ERP of mouse tracking [adapted from Hill (2014)]. The blue and yellow curves represent the activity of the neuronal unit oscillator sensitive to AT's target (Equation 3) and non-target (Equation 4). The red and purple curves represent the activity of the neuronal unit oscillator sensitive to the PT's speed (Equation 5) and direction (Equation 6). The green dashed curve demonstrates the AT stimuli's onset and duration. The black dashed line shows the effective threshold that activates the related unit [λ_trg_ = λ_ntrg_ = λ_S_ = λ_D_ = 0.2; A_trg_ = A_ntrg_ = A_S_ = A_D_ = 1; B_PT_ = B_AT_ = 1; Y_trg_ (0) = Y_ntrg_ (0) = Y_S_ (0) =Y_D_ (0) = 0.5].

Another effective factor is similarity that can generate higher effectiveness in the activity results, as shown in Figure 10.

According to Figure 10A, closing the frequency of non-target to target in AT leads the non-target unit to qualitatively behave similarly to the target unit as a result of facilitated synchronization. If the target unit frequency was indicated close to that of the non-target, then both features would be treated as non-targets, as expected. Therefore, if the features could oscillate together to a greater extent, their activation would become easier, with a lower attentional level. Nonetheless, if one of them is not desired, this similarity can cause difficulty for higher control levels to reduce it (Baghdadi et al., 2019). The RMS value shows more activity amplitude in the increased similarity of PT's features than those of AT. That may be because of the lower frequency of PT's features. Also, the variability of increased similarity of PT features is higher than AT's. Figure 10B shows how PT features can oscillate together with more similarities in their neuronal units. If the oscillators of PT's features were indicated to be closer together, then their synchronization would become easier, and the activity of the units would become enhanced. In this case, the top-down controller would have an easier task to perform, as this similarity can help the system to treat both units as one. Figure 10C shows the ERP of the central area of the brain during the mouse tracking task (Hill, 2014). The more the frequencies get close in the simulation, the more correspondences would appear to the ERP signal. Therefore, this indicates that the D and S features' more synchronization together would make a more actual brain activity signal of tracking and make the task closer to becoming automatic (Pashler, 1994; Annac et al., 2019).

## Discussion

A behavioral black box bottom-up attention model is proposed in this study. The model is based on neuronal oscillatory behavior, shown as the neuronal unit's activity presented by the Van der Pol oscillator. Additionally, effects of different levels of difficulties of AT and PT output under ST and DT conditions were examined considering the features of behavior outputs, such as RTs, AT errors, hesitation, and PT errors.

According to the statistical results, AT performance is more affected when the PT becomes harder (HPT) than the PT performance itself. The participants' attention is more involved in generating the correct output in the AT response as opposed to the PT task performance. Even when the PT becomes more difficult, the performance continues at an acceptable output feature rate. This can also be seen in the model's results. It should be noted that the related feature parameters are assumed to characterize the selective or movement feature and interact with the coupling of cortical neural units. The interaction is assumed to be directly effective, and the delays in intermediate interactions between cortical and subcortical areas have not been applied. This is consistent with previous studies that have assumed a bidirectional architecture for a bottom-up model in selective auditory attention (Trenado et al., 2009).

Figure 4 shows a situation in which no PT is running, and there is only one AT. Figure 5 demonstrates PT performance with a consistent behavior compared to the single AT. This behavior indicated that the bottom-up system synchronizes in the beginning of the continuous task, so the eyes could explore salient areas of the screen where there are potentially relevant features (Lungarella and Sporns, 2005). When both activate, as shown in Figure 6, the peak of activity rises higher, and the non-target becomes lower through the contribution of the coupling parameter. This was expected mathematically because of the higher difference of non-target frequency compared to the frequency of the other three features. Nevertheless, the interpretation of this behavior explains the component of the attention system. Since the entire model was designed for involuntary attention allocation, it appears that the resonance of the features is a contributional factor to the bottom-up filtering of salient stimuli (Knudsen, 2007). This may suggest that if there are more features with close frequencies, then bottom-up attention mechanisms could boost the manipulation of the initial task's knowledge (Ognibene et al., 2010). As shown in Figure 6, the bottom-up attentional system with matching initial information prevented the non-target from exploring using the units. A comparison of RMS values in Figure 4 shows that the performance reacted more to the S and target features (1.58 and 1.76, respectively) when the DT condition began. However, PT performance becomes synchronized after a short period of desynchronization. The consistency of movement features in PTs is evident in all Figures. Any change in amplitude and similarity results in slight asynchrony and subsequent fast synchronization. One interpretation associates this behavior with the non-zero use of attentional source. In other words, AT is receiving attention only during the application of a stimulus, while PT receives attention in all sequences.

**Figure 11:**
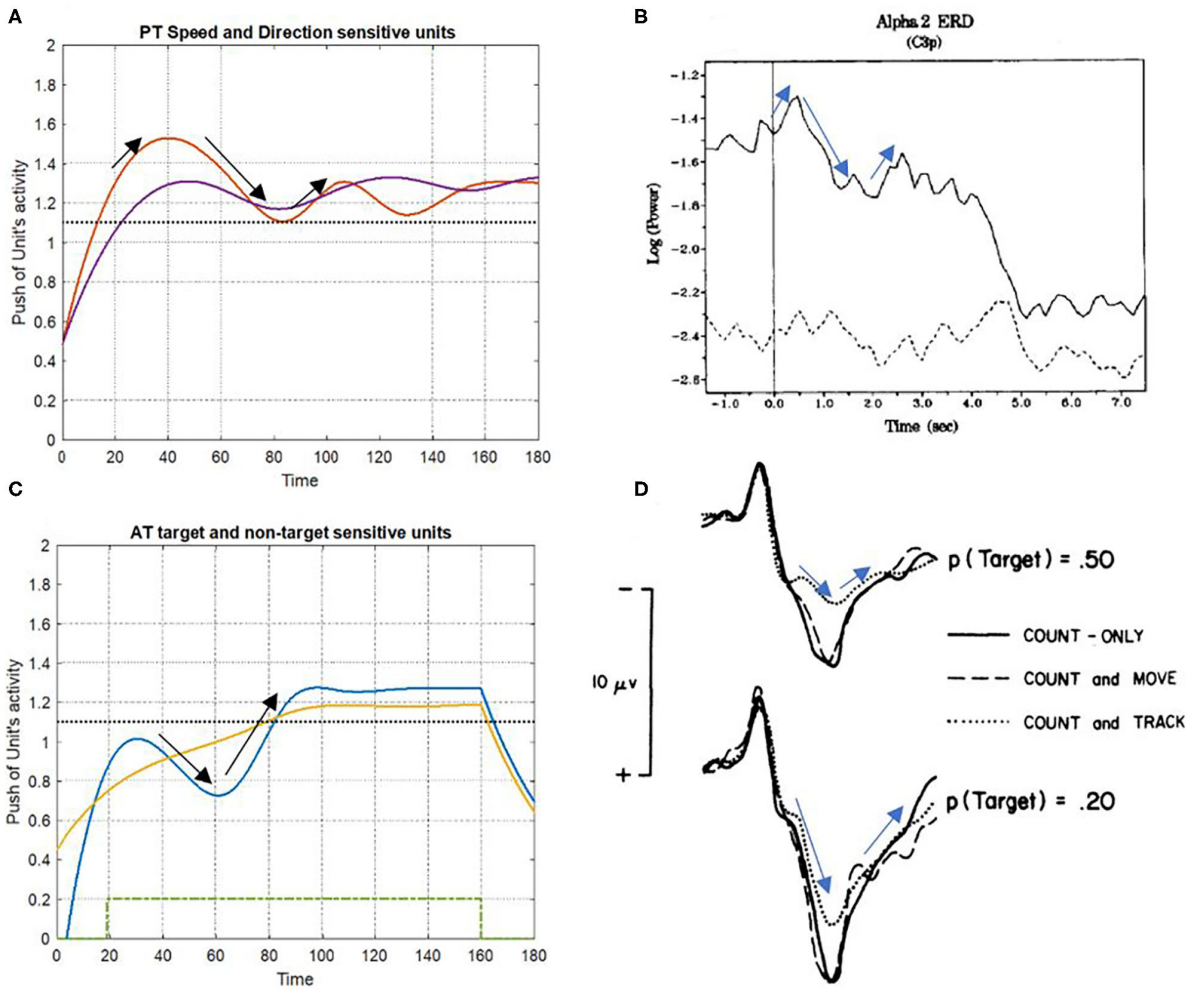
Consistency of simulation results with event-related synchronization (ERS) and event-related desynchronization (ERD) signals. **(A,C)** Show the simulation results of PT and AT features under DT conditions, respectively. The ERD and ERS signals have appeared in **(B,D)**, a multi-task condition with different difficulties [adapted from Fournier et al. (1999)]; and Da discrete DT condition [adapted from Isreal et al. (1980)].

The results of event-related synchronization (ERS) and event-related desynchronization (ERD) were used to compare the simulation findings against the brain activity unit. The alternation of brain activity under ST and DT conditions and the simulation results of the bottom-up attention system are found to be significantly similar to each other, as depicted in Figure 11. Notably, certain irregularities on the results that distinguish them from the real recorded brain activity can be associated with the fact that the proposed model does not include top-down attention and would not completely match the signals of brain activity.

The effect of the coupling parameter is illustrated in Figure 8. Under DT conditions with regular coupling between features and non-target frequency, intended to be considerably different from the target features, the bottom-up attention is capable of reducing non-target activity. However, when the coupling parameter was increased, the non-target activity exceeded the threshold. The reduction in non-target activity may have resulted from the role of the top-down attention system (Baghdadi et al., 2019). It has been found in previous studies that despite the top-down attention that is deteriorating at all levels of a disease, the bottom-up attentional system shows deficits only at the severe level of neurodegenerative diseases like Parkinson's' disease, because the retinal effect occurs in the high severity of the disease (Flowers and Robertson, 1995).

Effects of increasing the strength of input signals are shown in Figure 9. When AT strength increases, PT features demonstrate enhanced activity, which is also the case for AT activity, illustrated in Figure 9. Increasing the strength of both AT and PT features is similar to boosting the bottom-up attentional system to synchronize eatures. The difference among the strength results is very similar to the performance of brain activity of children with ADHD, as explained in the Results section (Manicolo et al., 2017). As shown in Figure 9, the pattern of oscillation and RMS value shows that the most reaction of activity is when the strength of both AT and PT increases, so with lack of signal strength, synchronization effectiveness lowers like the lower synchronization that leads to lower activity and attentional deficits in ADHD. Therefore, it can be concluded that enhancing the attention paid to one task generates higher awareness of the other task.

The increased similarity between the target and non-target shown in Figure 10 indicates the importance of initial decision regarding the frequency of units. In case a predefined task is non-existent with no targets and no feature coupling, then the similarity approaches zero, which can be observed in performance behavior. In addition, Figure 10 shows how a target unit would behave in the absence of similarity with PT feature coupling. According to the RMS value, more similarity leads to increase in activity of most attentional-demanding units, and this ends to consume the optimum energy for the whole procedure. If two PT features resonated with more similar frequencies, then the task would be performed with absolutely no difficulty. This may suggest the influence of skills and learning of PT. As depicted in Figure 10, increased similarity increases synchronization, and better synchronization leads to better performance, which is the purpose of learning, and more skills. It suggests that automatic performances are expected to have behaviors like close similarities of units (Yogev et al., 2005; Ulrich et al., 2015; Annac et al., 2019). The more units oscillate together, the more unity happens in task features and becomes an automatic task that is performed and learned.

The stability of the PT behavior under all conditions was not found to be consistent with the results of hesitation rate or even with the existence of hesitation in the results. This possibly suggests that the occurrence of hesitation can be associated with higher levels of attention, such as top-down attention control system, competitive selective attention, and the working memory system, which are voluntary features of the attention system. Given this explanation, hesitation cannot result from stimulus perception stage interference, a finding consistent with those of previous studies (Netick and Klapp, 1994; Klapp et al., 2019). It is also not the result of a startling AT occurrence (Netick and Klapp, 1994), considering the absence of a considerable activation drop observed in PT following the onset of the stimulus.

In the proposed model, synchronization was performed to create different behavior conditions. To this aim, parameters, such as strength, similarity, and coupling between features, were manipulated to create different unit behaviors.

## Conclusion

This study used a behavioral black-box model to investigate a discrete-continuous DT procedure. The possible effect of synchronization and desynchronization of features is shown to improve task performances. The model results were based on stimuli featuring neuronal units; the target and non-target features were defined for AT while the speed and direction features were used for continuous PT. The model's results showed that the RT of AT has a great correlation (84%) with the recorded RTs, while the PT's results showed no appearance of the hesitation factor (26% correlation with hesitation's variability). Along with comparing the results with figures from observations of ERP- and ERD-related studies, we can conclude that some aspects of RT's causes can appear in the stimulus detection and comprehension sections (occipital and parietal areas of the brain); however, no aspect of hesitation cause was observed to be situated in these areas. It was shown that the hesitation interference in PT could not have been due to stimulus perception, and that it pertains to higher cognitive attentional levels.

The model also demonstrated some activation pattern predictions in the results based on manipulation of the coupling parameters, strength, and similarity factors of the continuous task, and discrete task features. It was revealed that incrementing the coupling and strength factor can enhance bottom-up attentional system execution. An increment in similarity factor would lead to the performance of a more automatic task. However, there exist certain gaps in the procedure that indicate the importance of the role of the top-down attentional system. One of these gaps, as mentioned earlier, is the occurrence of hesitation, which does not appear in our proposed model's results. Adding the top-down model to the structure could solve this matter based on hesitation. Another gap would be the lack of ERD signals of the participants to compare with the model results. However, we compared the results of our simulations with other research reports, such as Fournier et al. (1999), Hill (2014), and Cowley et al. (2020). The model predicts the behavior of severe level of Parkinson's disease in decrement of coupling parameter representing the connectivity factor between units that represent the pattern of the severe level of parkinsonian in patients, and reduction of the strength factor would bring out the pattern that is representative of the behavior of ADHD. To improve the model, we suggest adding top-down attention as another unit to oscillate and couple with features; subsequently, the entire attentional system would be available to proceed.

## Data Availability Statement

The original contributions presented in the study are included in the article/supplementary material, further inquiries can be directed to the corresponding author.

## Ethics Statement

The studies involving human participants were reviewed and approved by Department of Psychology, Martin-Luther-University Halle-Wittenberg. The patients/participants provided their written informed consent to participate in this study.

## Author Contributions

MS contributed to conceptualization, methodology, software, formal analysis, investigation, data acquisition, writing of the original draft, and writing, reviewing, and editing the manuscript. MA-P contributed to conceptualization, methodology, reviewing and editing the manuscript, and supervision. FT contributed to conceptualization, methodology, and supervision. All authors contributed to the article and approved the submitted version.

## Conflict of Interest

The authors declare that the research was conducted in the absence of any commercial or financial relationships that could be construed as a potential conflict of interest.

## Publisher's Note

All claims expressed in this article are solely those of the authors and do not necessarily represent those of their affiliated organizations, or those of the publisher, the editors and the reviewers. Any product that may be evaluated in this article, or claim that may be made by its manufacturer, is not guaranteed or endorsed by the publisher.

## Footnotes

^1^Prof. Torsten Schubert: Professor of general psychology institute at the Martin Luther University Halle-Wittenberg.

^2^The path that the target of pursuit-tracking task is moving along that is established based on a function like sum of sinusoidal function that is called “force function”.

^3^Frame per second.

^4^Root mean square.

^5^Event-related potential (ERP) is the signal extracted from brain activity in response to a specific sensory or motor-cognitive event (Myers et al., 2014).

## Abbreviations

DT: Dual Task
ST: Single Task
RT: Response Time
H: Hesitation
AT: Auditory Task
PT: Pursuit Tracking
trg: target
ntrg: non-target
S: Speed
D: Direction
Seq: Sequence
SRT: Simple Response/Reaction time
CRT: Choice Response/Reaction time
EPT: Easy Pursuit Tracking
HPT: Hard Pursuit Tracking.
